# Heat shock protein 70-2 (HSP70-2) overexpression in breast cancer

**DOI:** 10.1186/s13046-016-0425-9

**Published:** 2016-09-22

**Authors:** Nirmala Jagadish, Sumit Agarwal, Namita Gupta, Rukhsar Fatima, Sonika Devi, Vikash Kumar, Vaishali Suri, Rajive Kumar, Vitusha Suri, Trilok Chand Sadasukhi, Anju Gupta, Abdul S. Ansari, Nirmal Kumar Lohiya, Anil Suri

**Affiliations:** 1 Cancer Microarray, Genes and Proteins Laboratory, National Institute of Immunology, Aruna Asaf Ali Marg, New Delhi, 110 067 India; 2 Department of Pathology, All India Institute of Medical Sciences, New Delhi, 110029 India; 3 All India Institute of Medical Sciences, Institute of Rotary Cancer Hospital, New Delhi, 110029 India; 4 Mahatma Gandhi Medical College and Hospital, Jaipur, 302022 India; 5 Department of Pathology, NMC Imaging and Diagnostic Centre, Vidyasagar Institute of Mental Health and Neuro-Sciences, New Delhi, 110065 India; 6 Centre for Advanced Studies, Department of Zoology, University of Rajasthan, Jaipur, 302 004 India

**Keywords:** HSP70-2, Breast cancer, Gene silencing, Apoptosis, Tumor growth

## Abstract

**Background:**

Breast cancer is one of the leading cause of cancer-related deaths in women worldwide and increasing rapidly in developing countries. In the present study, we investigated the potential role and association of HSP70-2 with breast cancer.

**Methods:**

HSP70-2 expression was examined in 154 tumor and 103 adjacent non-cancerous tissue (ANCT) specimens and breast cancer cell lines (MCF7, BT-474, SK-BR-3 and MDA-MB-231) by RT-PCR, quantitative-PCR, immunohistochemistry, Western blotting, flow cytometry and indirect immunofluorescence. Plasmid driven short hairpin RNA approach was employed to validate the role of HSP70-2 in cellular proliferation, senescence, migration, invasion and tumor growth. Further, we studied the effect of HSP70-2 protein ablation on signaling cascades involved in apoptosis, cell cycle and Epithelial-Mesenchymal-Transition both in culture as well as in-vivo human breast xenograft mouse model.

**Results:**

HSP70-2 expression was detected in majority of breast cancer patients (83 %) irrespective of various histotypes, stages and grades. HSP70-2 expression was also observed in all breast cancer cells (BT-474, MCF7, MDA-MB-231 and SK-BR-3) used in this study. Depletion of HSP70-2 in MDA-MB-231 and MCF7 cells resulted in a significant reduction in cellular growth, motility, onset of apoptosis, senescence, cell cycle arrest as well as reduction of tumor growth in the xenograft model. At molecular level, down-regulation of HSP70-2 resulted in reduced expression of cyclins, cyclin dependent kinases, anti-apoptotic molecules and mesenchymal markers and enhanced expression of CDK inhibitors, caspases, pro-apoptotic molecules and epithelial markers.

**Conclusions:**

HSP70-2 is over expressed in breast cancer patients and was involved in malignant properties of breast cancer. This suggests HSP70-2 may be potential candidate molecule for development of better breast cancer treatment.

**Electronic supplementary material:**

The online version of this article (doi:10.1186/s13046-016-0425-9) contains supplementary material, which is available to authorized users.

## Background

Breast cancer is the second leading cause of cancer related deaths among women worldwide [1]. Breast cancers are mostly adenocarcinomas which include invasive type such as infiltrating ductal carcinoma (IDC) and non-invasive type, Ductal Carcinoma in-situ (DCIS) [2]. IDC is the most common subtype of breast cancer accounting for 75–80 % of all the cases diagnosed [3]. Breast cancer incidence is highest in developed countries and increasing rapidly in developing countries due to lack of adequate medical support and infrastructure [4]. Therefore, there is a need to characterize a tumor-associated molecule for early detection of breast cancer and for identifying a novel therapeutic target for better cancer treatment.

Heat shock family of proteins (HSPs) is proposed to play pivotal role in breast tumor development owing to their intrinsic molecular chaperone properties [5]. HSPs are mainly involved in protein folding, assembly of multiprotein complexes, protein transport and protein targeting for lysosomal degradation [5]. Few of the HSPs have been reported in breast cancer which includes HSP27, HSP90 and HSP70 [6–8]. HSP70 family has eight members with high homology but different cellular localization and biological functions [8]. HSP70-2, a new member of cancer testis (CT) antigen family and an also an important member of HSP70 family has been found to be associated with various malignancies [8–11]. However, the clinical relevance and association of HSP70-2 expression in IDC specimens have not been investigated so far.

Our earlier studies have shown a close association of HSP70-2 with cellular proliferation, migration, invasion and tumor growth in urothelial [9] and cervical cancer [10]. In the present study, we have investigated the possible association of HSP70-2 mRNA and protein expression with various stages, grades and histotypes of breast cancer patients. In addition, we have examined the putative role of HSP70-2 in apoptosis, cell cycle arrest and epithelial-mesenchymal transition (EMT) in-vitro and in-vivo xenograft mouse model using gene silencing approach. Here we provide evidence that HSP70-2 expression is associated with IDC histotype of breast cancer. We also report that HSP70-2 plays an important role in cellular growth, migration and invasion of breast cancer cells and tumor growth of breast cancer xenograft. At molecular level, we show that HSP70-2 depletion resulted in up-regulation of caspases, pro-apoptotic molecules, cyclin dependent kinase (CDK) inhibitors and epithelial markers, and down-regulation of anti-apoptotic molecules, cyclins, CDKs and mesenchymal markers. These results collectively suggest that HSP70-2 could be used as a candidate for developing a novel therapeutic in breast cancer management.

## Methods

### Patient samples

The tumor specimens from 154 patients and 103 matched available adjacent non-cancerous tissue (ANCT) specimens were collected during surgical procedure at All India Institute of medical Sciences (AIIMS), New Delhi; Mahatma Gandhi Medical Hospital, Jaipur. All tumor specimens were collected from new patients who had not undergone treatment before surgery (study period 2008–2014). Details of specimens are illustrated in Table 1. The study was conducted as per the Ethical Committee approval obtained from AIIMS, Mahatma Gandhi Medical Hospital, Jaipur and National Institute of Immunology, New Delhi. The duly signed consent forms were obtained from the patients prior to the study.

**Table 1:** HSP70-2 expression (RT-PCR/IHC) and clinico-pathologic characteristics of breast cancer

Pathologic and clinical features	RT-PCR (%)	IHC (%)	*P* value
All tumors	128/154 (83)	128/154 (83)	0.569
Histotypes			
DCIS	8/8 (100)	8/8 (100)	
IDC	116/139 (83.4)	116/139 (83.4)	
ILC	4/5 (80)	4/5 (80)	
DCIS + IDC	2/2 (100)	2/2 (100)	
Tumor stages (IDC)			
Stage I	3/3 (100)	3/3 (100)	
Stage II	68/85 (80)	68/85 (80)	0.388
Stage III	39/45 (86.7)	39/45 (86.7)	0.343
Stage IV	6/6 (100)	6/6 (100)	0.341
Stages			
Early Stages (I + II)	71/88 (80.7)	71/88 (80.7)	0.248
Late Stages (III + IV)	45/51 (88.2)	45/51 (88.2)	
Grades			
Grade 1	62/69 (89.8)	62/69 (89.8)	
Grade 2	39/52 (75)	39/52 (75)	0.029*
Grade 3	15/18 (83.3)	15/18 (83.3)	0.468
Lymph node involvement			
Positive	40/51 (78.4)	40/51 (78.4)	
Negative	76/88 (86.4)	76/88 (86.4)	

*p < 0.017, Bonferroni correction value

### Cell lines

Four breast cancer cell lines of different hormone receptor profile, MCF7 (luminal-A, ER^+^PR^+^Her2^−^), BT-474 (luminal-B, ER^+^PR^+^Her2^+^), SK-BR-3 (HER2 overexpressing, ER^−^PR^−^Her2^+^) and MDA-MB-231 (highly metastatic basal, triple-negative ER^−^PR^−^Her2^−^) were procured from American Type Culture Collection (ATCC, Manassas, VA). All the cells were cultured in recommended medium under standard conditions. Human normal mammary epithelial cells (HNMEC’s) were purchased and maintained according to manufacturer’s directions (Gibco, Life Technologies Corporation, Carlsbad, CA).

### RT-PCR, Real time-PCR analysis, Western blotting, flow cytometric analysis and immunofluorescence, immunohistochemistry

HSP70-2 mRNA and protein expression was examined in tumor specimens and all four breast cancer cell lines along with HNMEC’s as detailed in Additional file 1 and Methods section.

### Immunoreactivity score (IRS)

Immunoreactivity score (IRS) was calculated as a percentage of cells expressing HSP70-2 protein. For determining the IRS, the tissue section slides were independently reviewed by two senior pathologists. More than 500 cells were counted from five random fields at 400× magnifications. Specimens showing >10 % HSP70-2 positive cells were considered as positive immuno-reactive specimens.

### Validation of shRNA targets against HSP70-2

Four shRNA constructs against HSP70-2 along with scrambled negative control NC shRNA were procured from Super Array (Frederick, MD, USA) as detailed earlier [9]. The transient transfections were carried out in MCF7 and MDA-MB-231 cells using lipofectamine (Invitrogen Life Technologies Corporation, USA) and HSP70-2 knockdown efficiency was determined by Real-time PCR and Western blotting as described in Additional file 1 and Methods section.

### Cellular proliferation analysis, cell viability and colony formation assay

Cellular proliferation, viability and colony forming assay was carried in HSP70-2 shRNA3, shRNA4 and NC shRNA transfected MCF7 and MDA-MB-231 cells out as detailed in Additional file 1 and Methods section.

### Cell cycle analysis

The HSP70-2 shRNA3, shRNA4 and NC shRNA transfected MDA-MB-231 cells were fixed in ethanol and stained with propidium iodide with RNaseA as described earlier [12]. The cells were analyzed by flow cytometry on BD-VERSA (BD Biosciences, California, USA).

### Cellular senescence assay

HSP70-2 shRNA3, shRNA4 and NC shRNA transfected MDA-MB-231 cells were stained with X-gal using Senescence kit (Sigma-Aldrich, St. Louis, MO, USA) as per manufacturer’s instructions. The images were captured using Nikon Eclipse E400 microscope (Nikon, Fukok, Japan).

### Scanning electron microscopy

MDA-MB-231 and MCF7 cells were treated with lipofectamine, DMSO, HSP70-2 shRNA3, shRNA4 and NC shRNA. The cells were processed as described earlier [13]. The images were captured using electron microscope (EVO LSM10 Zeiss, Germany) at 20 kV using SmartSEM software in central microscopic facility.

### TUNEL assay

DNA fragmentation due to shRNA treatment was studied using Apo-BrdU-Red In-situ DNA fragmentation assay kit (Biovision, California, USA). HSP70-2 shRNA3, shRNA4 and NC shRNA transfected MDA-MB-231 cells were processed as described earlier [13]. The cells were analyzed at 576 nm using BD-FACS VERSA. (BD Biosciences, California, USA).

### M30 assay

HSP70-2 shRNA3, shRNA4 and NC shRNA transfected MDA-MB-231 cells were fixed with methanol and M30 assay was carried using M30 cytoDEATH antibody (Roche Diagnostics, GmBH, Mannheim, Germany). The cells were analyzed using BD-FACS VERSA. (BD Biosciences, California, USA).

### Chromatin condensation assay

HSP70-2 shRNA3, shRNA4 and NC shRNA transfected MDA-MB-231 cells were harvested, washed with PBS and stained with 5 μg/ml of DAPI for 3 min at 37 °C. The level of chromatin condensation was determined by flow cytometry using BD-FACS VERSA (BD Biosciences, California, USA).

### AnnexinV staining

To study the effect of HSP70-2 shRNA3 and shRNA4 on apoptosis compared to NC shRNA, MDA-MB-231 cells were stained with annexinV using annexinV-PerCP-Cy5-5-A staining kit (Biovision, CA, USA) and assay carried out as described earlier [13].

### Mitochondrial membrane potential

MDA-MB-231 cells transfected with NC shRNA, HSP70-2 shRNA3 and shRNA4 were stained with 500 nM tetramethyl rhodamine ethyl ester (TMRE Assay kit, Abcam, Cambridge, United Kingdom) for 2 min at 37 °C. Mitochondrial membrane potential was analyzed using BD-FACS VERSE (BD Biosciences, California, USA).

### Cell migration, invasion and wound healing assay

Cell migration, invasion and wound healing ability of HSP70-2 shRNA3, shRNA4 and NC shRNA transfected MCF7 and MDA-MB-231 cells were assessed as described in Additional file 1 and Methods section.

### In-vivo xenograft studies

Athymic nude mice (National Institute of Immunology [NII], National Institutes of Health, [S] nu/nu) were used for the xenograft studies. Mice were injected subcutaneously with 5 × 10^6^ MDA-MB-231 cells. The experiment was conducted as described in Additional file 1 and Methods section.

### Statistical analysis

The statistical analysis was performed using SPSS 20.0 statistical software package (SPSS Inc, Chicago, IL, USA). Pearson’s Chi-Square test was performed among various grades and stages. *P*-values ≤ 0.017 among grades and *P*-value ≤ 0.013 were considered as statistically significant after applying a Bonferroni correction for multiple comparisons. Based on immuno-reactivity score (IRS), the IDC specimens were divided in; Group I including specimens with >50 % cells expressing HSP70-2 protein, Group II; including specimens with <50 % cells expressing this protein. The statistical difference of HSP70-2 protein expressing cells in group I and group II was determined by Mann Whitney test. Statistical comparisons of mean values in cell line study were performed using Student’s t-test. A *P*-value <0.05 was considered statistically significant. Data is expressed as mean ± standard error of three independent experiments in triplicates in in-vitro assays.

## Results

### HSP70-2 is overexpressed in clinical samples of breast tumors

**Fig. 1:**
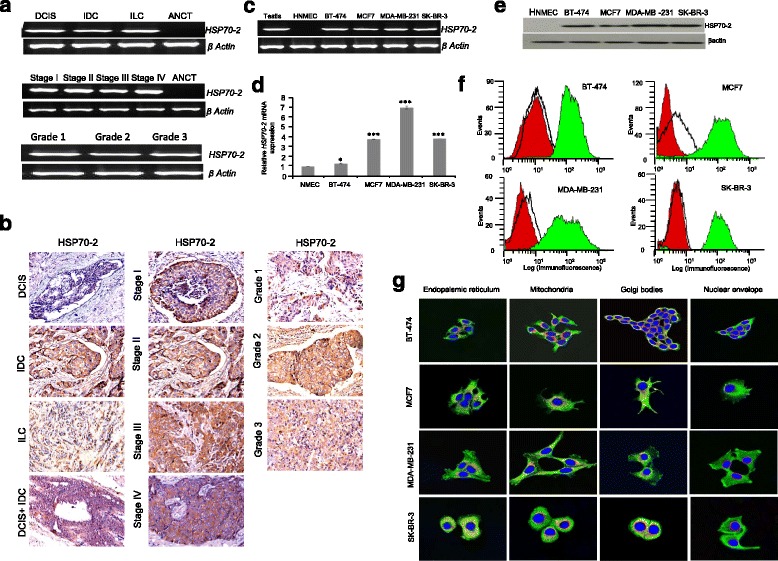
HSP70-2 gene and protein expression in clinical specimens and breast cancer cell. **a** Representative gel picture of RT-PCR analysis shows HSP70-2 gene expression in different histotypes (DCIS, IDC, ILC), stages (I-IV) and grades (1–3) of breast cancer specimens. **b** Representative images of IHC analysis of tissue sections show HSP70-2 protein expression (chocolate brown reactivity) in different histotypes (DCIS, IDC, ILC), stages (I-IV) and grades (1–3) of breast cancer specimens DCIS: Ductal Carcinoma in-situ, IDC: Infiltrating Ductal carcinoma and ILC: Infiltrating Lobular Carcinoma. Objective: ×20, Magnification: ×200. **c** RT-PCR analysis shows *HSP70-2* gene expression in breast cancer cells: BT-474, MCF7, MDA-MB-231 and SK-BR-3. However, no *HSP70-2* gene expression was observed in human normal mammary epithelial cells (HNMEC’s). Testis was used as positive control and β-actin as loading control. **d** Histogram shows the qPCR analysis depicting higher expression of *HSP70-2* gene in breast cancer cells as compared to HNMECs. **e** Western blot analysis shows HSP70-2 protein expression in all breast cancer cells compared to no expression in HNMECs. **f** Flow cytometric analysis showed surface localization (displacement of fluorescence intensity on X-axis) of HSP70-2 protein in all breast cancer cells. Red peak shows unstained population, black peak shows cell population stained with control IgG antibody and green peak shows cell population probed with anti-HSP70-2 antibody. **g** IIF depicts co-localization (yellow-orange staining) of HSP70-2 in endoplasmic reticulum, mitochondria and Golgi bodies. However, no co-localization was observed in nuclear envelope. Nucleus is stained with DAPI. Objective: ×63, Magnification: ×630

The expression of HSP70-2 mRNA and protein was examined in breast clinical cancer specimens by qRT-PCR and IHC respectively. *HSP70-2* gene expression was detected in 83 % (128/154) of total breast cancer tissue specimens irrespective of clinicopathological features of breast cancer tissue specimens including histotypes, stages and grades but not in ANCT samples (Fig. 1a, Table 1). Congruent with RT-PCR data HSP70-2 protein expression was also detected in 83 % (128/154) tissue specimens (Fig. 1b) but not in matched ANCT (Additional file 2: Figure S1a, c). Notably, HSP70-2 expression was observed in 100 % of (8/8) DCIS, 83.4 % (116/139) of IDC, 80 % (4/5) of ILC and 100 % (2/2) of DCIS + IDC specimens. Furthermore, HSP70-2 expression was found in 100 % (3/3) of stage I, 80 % (68/85) of stage II, 86.7 % (39/45) of stage III and 100 % (6/6) stage IV of IDC histotypes of tissue specimens. HSP70-2 expression was detected in 89.8 % (62/69) of grade 1, 75 % (39/52) of grade 2 and 83.3 % (15/18) of grade 3 IDC specimens (Table 1). In addition, 80.4 % (41/51) of IDC specimens were found positive for HSP70-2 expression that had lymph node involvement (stage III and IV), whereas, 86.4 % (76/88) specimens with negative lymph node involvement (stage I and II) showed HSP70-2 expression (Table 1).

Based on immuno-reactivity score (IRS), the IDC specimens were divided in two groups as shown in Additional file 2: Figure S1d. Group I included specimens with >50 % cells expressing HSP70-2 protein, whereas, Group II included specimens with <50 % cells expressing this protein. Interestingly, number of patients (75.9 %, 88/116) expressing HSP70-2 was significantly higher (*P < 0.0001*) in Group I (IRS = 72.74 ± 1.34) compared to Group II patients (24.1 %, 28/116, IRS = 28.07 ± 1.89, Additional file 2: Figure S1d) irrespective of stages, grades and histotypes. Based on the HSP70-2 IRS score, after the Bonferroni correction for multiple comparisons, HSP70-2 protein expression were found to be significantly associated with grade 1 (63.71 ± 2.61) and grade 2 cases (63.74 ± 3.69, *P* < 0.029; Table 1). In addition, HSP70-2 protein expressing cells in patients with positive lymph node involvement (55.33 ± 3.44) and negative lymph node involvement (65.64 ± 2.56) was also observed.

### HSP70-2 is over-expressed in breast cancer cell lines

Since breast tumor samples showed overexpression of HSP70-2 mRNA as well as protein, we next examined its expression in four different breast cancer cell lines viz., BT-474, MCF7, MDA-MB-231 and SK-BR-3. As shown in Fig. 1c, *HSP70-2* mRNA expression was detected in all four breast cancer cells irrespective of their molecular phenotype but not in human normal mammary epithelial cells (HNMEC; Fig. 1c). There was higher *HSP70-2* mRNA expression in triple negative MDA-MB-231 (>7 fold; *P* < 0.001) cells, followed by MCF7 (>3fold; *P* < 0.001), SK-BR-3 (>3 fold; *P* < 0.001) and BT-474 (1.28 fold; *P* < 0.05) with respect to HNMEC (Fig. 1d). Further Western blot analysis confirmed the expression of HSP70-2 protein in in all cell lines (Fig. 1e) along with their surface localization (Fig. 1f). In addition, we also observed cytoplasmic presence of HSP70-2 in all these cells (Additional file 2: Figure S1e) with specific localization in endoplasmic reticulum, mitochondria and Golgi bodies but not with nuclear envelope (Fig. 1g).

### Down-regulation of HSP70-2 leads to reduced cellular proliferation, cell viability, colony forming ability of breast cancer cells

**Fig. 2:**
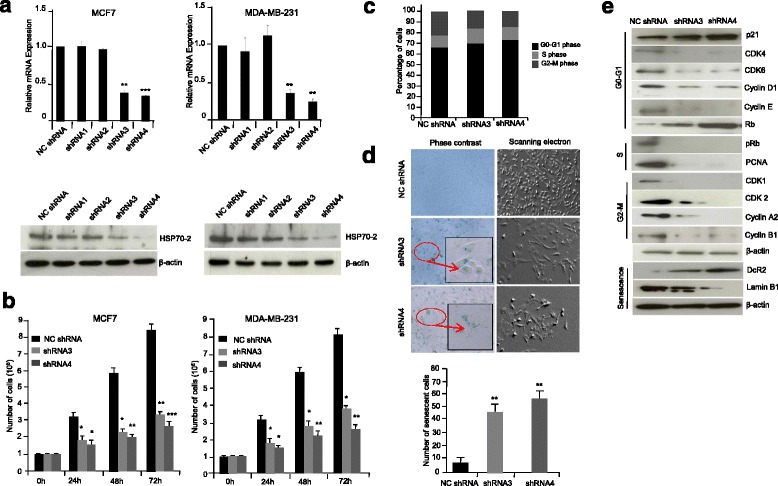
HSP70-2 protein ablation arrests cell cycle growth and induces cellular senescence in breast cancer cells. **a** Quantitative-PCR and Western blot analysis shows maximum HSP70-2 ablation with shRNA3 and shRNA4 transfected MCF7 and MDA-MB-231 cells compared to NC shRNA, shRNA1 and shRNA2 transfected cells. **b** Histogram depicts significant reduction in cellular proliferation after 24, 48 and 72 h in shRNA3 and shRNA4 transfected MCF7 and MDA-MB-231 cells compared to NC shRNA transfected cells. **c** Histogram depicts the accumulation of cells in G_0_/G_1_ phase in MDA-MB-231 cells transfected with shRNA3 and shRNA4 compared to NC shRNA. **d** Phase contrast (*left panel*) and SEM (*right panel*) images show onset of senescence as shown by β-galactosidase staining (*green color*) and flattened morphology, respectively in shRNA3 and shRNA4 transfected MDA-MB-231 cells compared to NC shRNA. Inset shows zoom image of selected region from the core. Histogram demonstrates percentage of cells observed under phase contrast microscopy images undergoing senescence when transfected with NC shRNA, shRNA3 and shRNA4. Phase contrast microscopy: Original magnification ×100, objective ×10. SEM: Magnification: ×700, WD = 6 mm, EHT = 20.00 kV. **e** Western blot analysis showed decreased expression of CDK4, CDK6, Cyclin D1, Cyclin E, pRb, PCNA, CDK1, CDK2, Cyclin A2, Cyclin B1, Lamin B1, while increased expression of p21, Rb, DcR2 in shRNA3 and shRNA4 transfected MDA-MB-231 cells compared to NC shRNA. The three independent experiments were carried out in triplicates. Data are represented as mean ± SEM. *P < 0.05, **P < 0.001, ***P < 0.0001

Interestingly we observed the overexpression of HSP70-2 in breast tumor as well as in breast cancer cell lines, therefore we next investigated its physiological relevance in oncogenic properties of cancer cells. Knock-down HSP70-2 expression was examined employing four HSP70-2 shRNA targets and scrambled shRNA (NC shRNA) in MCF7 and MDA-MB-231 cancer cells. Our qPCR and Western blot analysis clearly showed that HSP70-2 gene and protein expression was specifically down-regulated with HSP70-2 shRNA3 (*P* < 0.001; *P* < 0.002) and shRNA4 (*P* < 0.0001; *P* < 0.005) compared to HSP70-2 shRNA1, shRNA2 and NC shRNA (Fig. 2a) in MCF7 and MDA-MB-231 cells respectively. Therefore, in all subsequent in-vitro assays, HSP70-2 shRNA3 and shRNA4 targets were used in cell culture. Our cell proliferation studies revealed a significant reduction in cell count by 46.7 % (*P < 0.0001*) and 41.5 % *(P < 0.0001*) respectively in HSP70-2 shRNA3 and shRNA4 transfected MCF7 cells post 72h respectively as compared to NC shRNA transfected cells (Fig. 2b). Expectedly, MDA-MB-231 cells also showed reduction in cell count by 48.4 % (*P < 0.05*) in HSP70-2 shRNA3 transfected cells and 43.9 % (*P < 0.001*) in HSP70-2 shRNA4 transfected cells. Also, there was a significant reduction *(P < 0.001*) in cell viability of HSP70-2 shRNA3 and shRNA4 transfected MCF7 and MDA-MB-231 cells compared to NC shRNA transfected cells (Additional file 3: Figure S2a). Importantly, HSP70-2 depletion also resulted in a significant reduction in the colony forming ability of MCF7cells by 60.79 % (*P < 0.0001*) with HSP70-2 shRNA3 and 63.16 % (*P < 0.0001*) with HSP70-2 shRNA4 transfection (Additional file 3: Figure S2b). MDA-MB-231 cells also exhibited marked reduction of 60.01 % (*P < 0.0001*) and 63.87 % (*P < 0.0001*) in cells transfected with HSP70-2 shRNA3 and shRNA4 respectively (Additional file 3: Figure S2b). Thus, HSP70-2 seems to play an important role in cell proliferation, cell viability and tumorigensis.

### Knockdown of HSP70-2 results in cell cycle arrest and induces senescence

Next we investigated the role of HSP70-2 in cell cycle. We observed that down-regulation of HSP70-2 expression led to accumulation of MDA-MB-231 cells in G_0_/G_1_ phase in HSP70-2 shRNA3 and shRNA4 treated cells (Fig. 2c and Additional file 3: Figure S2c). Further, phase contrast microscopy of these cells showed enhanced β-galactosidase staining in HSP70-2 depleted MDA-MB-231 cells (green color; Fig. 2d), while scanning electron microscopy (SEM) images showed flattened phenotype of these cells compared to normal (Fig. 2d ). Besides, HSP70-2 depleted MDA-MB-231 cells showed increased onset of senescence (*P <* 0.001; Fig. 2d) as also evident from enhanced expression of senescence associated marker, Decoy receptor 2 (DCR2) and lamin B1 in these cells (Fig. 2e). Thus, these results suggested that depletion of HSP70-2 seems to initiate senescence process.

We further examined the status of molecules involved in cell cycle arrest. Our qPCR results in HSP70-2 depleted MDA-MB-231 cells revealed 1.6 fold up-regulation of *p21* expression (*P* < 0.019) with a concomitant reduction in *CDK1* (*P <* 0.007*)*, *CDK2* (*P <* 0.009*)*, *CDK4 (P* < 0.0003), *CDK6* (*P* < 0.006)*, cyclin B1* (*P* < 0.027), *cyclin D1* (*P* < 0.007) and *cyclin E* levels (*P* < 0.008; Additional file 3: Figure S2d). Further, Western blot analysis confirmed the over-expression of CDK inhibitors, p21 and decreased expression of cyclin D1, cyclin E, CDK4 and CDK6 and reduced expression of G_2_/M-phase CDK1, CDK2 and cyclin B1 and cyclin A2 in HSP70-2 depleted cells (Fig. 2e). We also observed decreased levels of phosphorylated Rb and corresponding accumulation of Rb due to G_0_/G_1_ arrest of the cells. As expected, proliferating cell nuclear antigen (PCNA) expression also decreased as shown in Fig. 2e. Collectively, these results suggested that G_0_/G_1_ arrest caused by ablation of HSP70-2 may be mediated through up-regulation of p21 and down-regulation of cyclins and their cognate kinases.

### HSP70-2 gene silencing initiates apoptosis

**Fig. 3:**
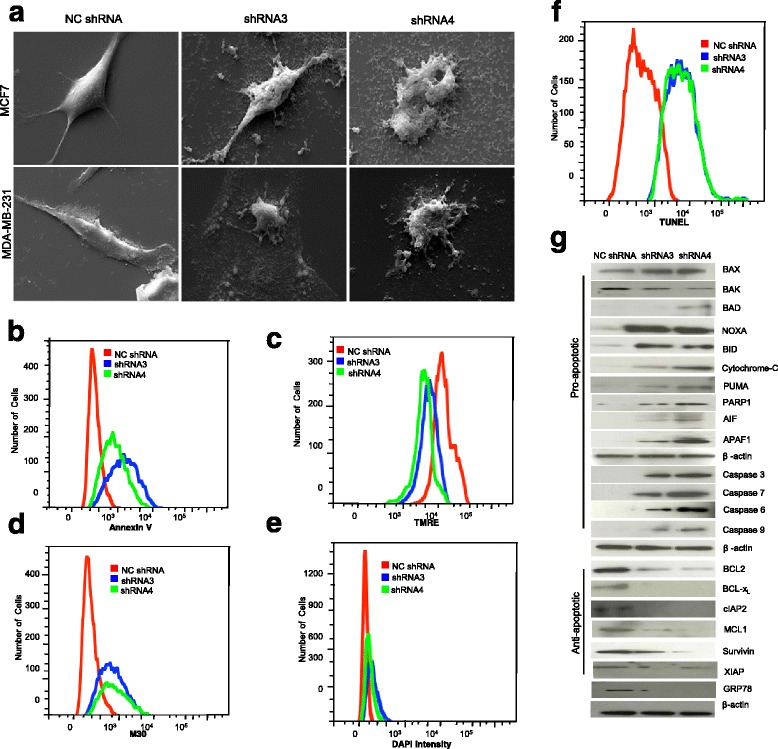
HSP70-2 depletion results in apoptosis of breast cancer cells. **a** Representative SEM images show onset of apoptosis characterized by membrane blebbing, holes and apoptotic bodies formation in shRNA3 and shRNA4 transfected MCF7 and MDA-MB-231 cells compared to NC shRNA transfected cells. Magnification: ×2000, WD = 6 mm, EHT = 20.00 kV. **b** Flow cytometric analysis shows increased number of MDA-MB-231 cells bound to annexinV when transfected with shRNA3 and shRNA4 compared to NC shRNA transfected cells. **c** Flow cytometric data demonstrates loss of mitochondrial membrane potential in MDA-MB-231 cells transfected with shRNA3 and shRNA4 compared to NC shRNA transfected cells. **d** Flow cytometric analysis depicts increased number of MDA-MB-231 cells expressing M30 on their surface when transfected with shRNA3 and shRNA4 compared to NC shRNA transfected cells. **e** FACS data shows increased DAPI intensity in MDA-MB-231 cells transfected with shRNA3 and shRNA4 compared to NC shRNA. **f** FACS analysis demonstrates increased apo-BrdU staining in shRNA3 and shRNA4 transfected MDA-MB-231 cells compared to NC shRNA transfected cells. **g** Western blot analysis shows up-regulation of pro-apoptotic (BAX, BAK, BAD, NOXA, BID, cytochrome-C, PUMA, PARP1, AIF, APAF1, Caspase 3, Caspase 6, Caspase 7, Caspase 9) molecules and down-regulation of anti-apoptotic molecules (BCL2, BCL-x_L_, cIAP2, MCL1, Survivin, XIAP, GRP78) in HSP70-2 depleted MDA-MB-231 cells. The three independent experiments were carried out in triplicates

The phenotypic changes associated with apoptosis were investigated by SEM in HSP70-2 depleted MCF7 and MDA-MB-231 cells. Post 24 h, both breast cancer cells exhibited early signs of apoptosis including cell shrinkage and blebbing, followed by the formation of apoptotic bodies in HSP70-2 shRNA3 and shRNA4 transfected cells (Fig. 3a). Post 48 h, most of the cells appeared to undergo apoptotic death. However, no phenotypic changes in the morphology of NC shRNA transfected (Fig. 3a) and lipofectamine treated cells were observed (Additional file 4: Figure S3a).

To elucidate the role of HSP70-2 in apoptosis, we analyzed several characteristic markers of apoptosis in HSP70-2 depleted cells. We analyzed annexinV staining to examine the externalization of phosphatidyl serine, an early sign of apoptosis. Our flow cytometry data showed marked increase in annexinV staining in MDA-MB-231 cells transfected with HSP70-2 shRNA3 and shRNA4 as compared to NC shRNA (Fig. 3b). We also determined changes in the mitochondrial membrane potential (MMP) of HSP70-2 depleted MDA-MB-231 cells using TMRE (tetramethyl rhodamine, ethyl ester) dye. Our data revealed loss of potential due to HSP70-2 shRNA treatment (Fig. 3c). Next, we investigated caspase cleavage by employing M30 assay and found higher population of M30 positive (Fig. 3d). To monitor the late apoptosis events, we also examined chromatin condensation by DAPI staining and observed a rise in the DAPI intensity in HSP70-2 depleted MDA-MB-231 cells (Fig. 3e). In addition, we examined DNA fragmentation by TUNEL assay and observed a marked increase in BrdU positive MDA-MB-231cells (Fig. 3f).

Analysis of pro-apoptotic gene expression by qPCR in HSP70-2 shRNA4 treated cells revealed a significant increase in the expression of *BID* (*P* < 0.003)*, caspase 6* (*P* < 0.033)*, caspase 7* (*P* < 0.008)*, caspase 9* (*P* < 0.012)*, PUMA* (*P* < 0.002) and *cytochrome-C* (*P* < 0.007) by 1.39, 1.83, 1.72, 1.89, 3.0 and 1.92 folds respectively. Whereas the expression of anti-apoptotic molecules including *BCL2* (*P* < 0.003), *BCL-x*_*L*_ (*P* < 0.045), *Survivin* (*P* < 0.009), *cIAP2* (*P* < 0.003), *XIAP* (*P* < 0.035) and *MCL1* (*P* < 0.007) was significantly inhibited (Additional file 4: Figure S3b). Further, Western blot analysis revealed increased expression of molecules involved in intrinsic pathway including caspase 3, caspase 6, caspase 7, caspase 9 and cytochrome-C (Fig. 3g). The levels of pro-apoptotic molecules including BID, BAD, BAK, BAX, NOXA, APAF1 and PUMA were also up-regulated in these cells. As expected, low level of expression of several anti-apoptotic molecules including BCL2, BCL-x_L_, Survivin, MCL1 and XIAP was observed (Fig. 3g). Interestingly, increased expression of AIF and PARP-1 in these cells suggested the activation of caspase-independent pathway under these conditions (Fig. 3g). Considering the important role of HSP70-2 in protein folding and degradation, activation of intrinsic apoptotic pathway due to HSP70-2 abrogation prompted us to investigate whether the ER stress could be the underlying cause of pro-apoptotic cell death. We did find decrease protein expression of ER chaperone protein, GRP78 in HSP70-2 depleted cells (Fig. 3g) suggesting its essential role in cell survival.

### HSP70-2 is essential for cellular motility, migration and invasion

**Fig. 4:**
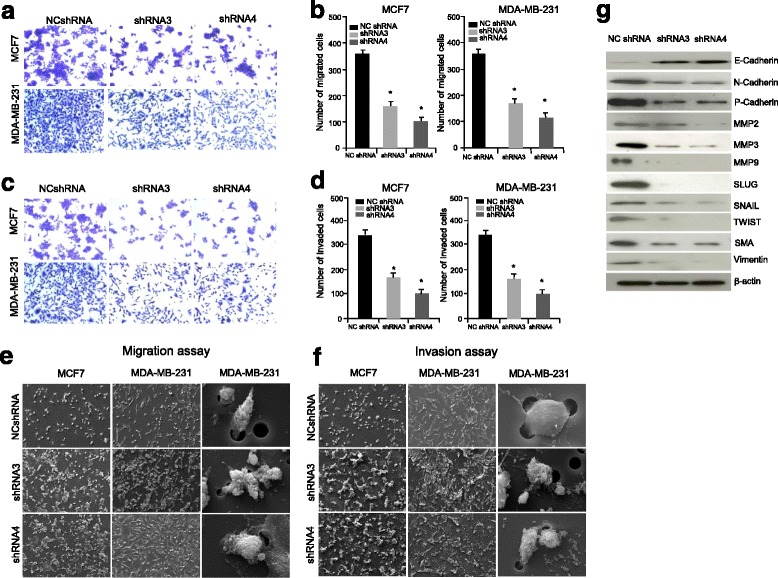
HSP70-2 ablation inhibits cellular motility of breast cancer cells. **a** Phase contrast microscopy images show difference in number of MCF7 and MDA-MB-231 cells migrating through the transwell-insert membrane when transfected with shRNA3, shRNA4 compared to NC shRNA. **b** Histogram demonstrates significant reduction in number of migratory MCF7 and MDA-MB-231 cells transfected with shRNA3 and shRNA4 compared to NC shRNA. **c** Phase contrast microscopy images depict reduced number of MCF7 and MDA-MB-231 cells invading through matrigel coated transwell insert membrane when transfected with shRNA3 and shRNA4 compared to NC shRNA. **d** Histogram shows significantly reduced number of shRNA3 and shRNA4 transfected MCF7 and MDA-MB-231 cells invading through insert as compared to NC shRNA. *P < 0.05. Data are represented as mean ± SEM. **e** SEM images show difference in the number of MCF7 (*first panel*) and MDA-MB-231 (*second panel*) cells migrating through transwell insert membrane when transfected with shRNA3 and shRNA4 compared to NC shRNA in migration. Magnification: ×700, WD = 6 mm, EHT = 20.00 kV. *Third panel* shows single MDA-MB-231 cell migrating through the transwell insert membrane. Magnification: ×4000, WD = 6 mm, EHT = 20.00 kV. **f** SEM images depict difference in the number of MCF7 (*first panel*) and MDA-MB-231 (*second panel*) cells invading through transwell insert membrane when transfected with shRNA3 and shRNA4 compared to NC shRNA. Magnification: ×700, WD = 6 mm, EHT = 20.00 kV. *Third panel* shows single MDA-MB-231 cell invading through the transwell insert membrane. Magnification: ×4000, WD = 6 mm, EHT = 20.00 kV. The three independent experiments were carried out in triplicates. **g** Western blot analysis demonstrates up-regulation of epithelial marker (E-Cadherin) and down-regulation of mesenchymal markers (N-Cadherin, P-Cadherin, MMP2, MMP3, MMP9, SLUG, SNAIL, TWIST, SMA, Vimentin) in HSP70-2 depleted MDA-MB-231 cells

Increased cellular motility, migration and invasion are distinguishing features of cancer cells. We studied transwell membrane assays to study the effect of ablation of HSP70-2 on migratory and invasive properties of MCF7 and MDA-MB-231cells. The cell migration assay exhibited a significant reduction *(P < 0.05)* in the migration of HSP70-2 shRNA3 and shRNA4 transfected cells compared to NC shRNA (Fig. 4a, b) with a concomitant loss of invasive ability through matrigel *(P < 0.05,* Fig. 4c, d). Further, the SEM images of transwell membranes confirmed reduced migration of these cells (Fig. 4e, f). In addition, wound healing assay also indicated reduced cellular motility under the conditions as compare to control cells (Additional file 5: Figure S4a).

Epithelial-Mesenchymal Transition (EMT) is considered to be a benchmark in cancerous growth. Therefore, we measured the mRNA expression of EMT markers in HSP70-2 depleted cells. As shown in Additional file 5: Figure S4b, there was an overall significant reduction in the mRNA levels of mesenchymal markers such as *N-Cadherin* (*P* < 0.0001)*, P-Cadherin* (*P* < 0.012)*, MMP2* (*P* < 0.0001)*, MMP3* (*P* < 0.004)*, SLUG* (*P* < 0.006)*, SNAIL* (*P* < 0.049)*, Vimentin* (*P* < 0.005) and *SMA* (*P* < 0.010). However the epithelial cell marker, *E-Cadherin* (*P* < 0.002), showed an increased expression of 2.74 fold (Additional file 5: Figure S4b). Further, Western blot analysis validated our qPCR data revealing down-regulation of SNAIL, SLUG and TWIST (EMT regulators) along with SMA, Vimentin, N-Cadherin, P-Cadherin, MMP2, MMP3 and MMP9 (Fig. 4g). As expected, there was increase in E-Cadherin expression (Fig. 4g). Thus, HSP70-2 seems to play an important role in cellular migration and invasion orchestrated via EMT pathway.

### Depletion of HSP70-2 causes reduced xenograft breast tumor growth in-vivo

**Fig. 5:**
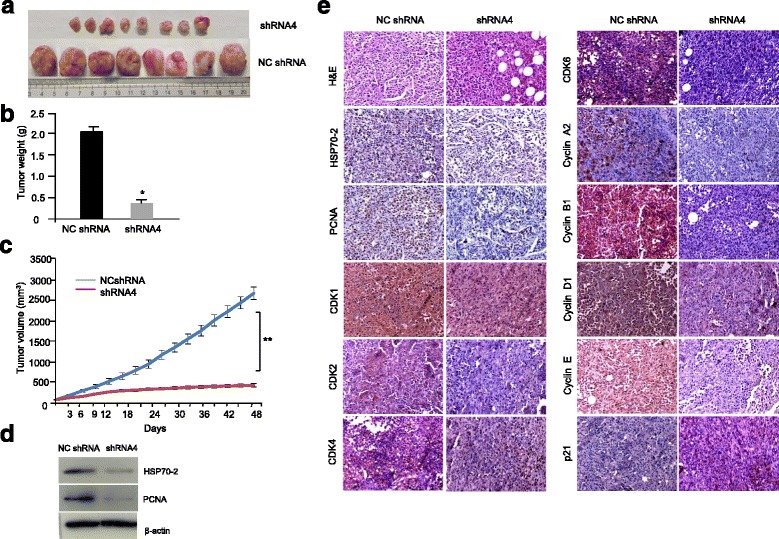
HSP70-2 shRNA treatment reduces tumor growth of human breast cancer xenograft. **a** Representative image shows reduced tumor size of the mice treated with shRNA4 (*n* = 8) compared to NC shRNA (*n* = 8). **b** Histogram shows significant difference in weight of tumor when treated with shRNA4 compared to NC shRNA. **c** Line graph shows significant difference in tumor volume when treated with shRNA4 compared to NC shRNA. **d** Western blot analysis depicts decreased expression of HSP70-2 and PCNA in shRNA4 treated tumor as compared to NC shRNA. **e** Representative images show IHC analysis of serial sections of shRNA4 and NC shRNA treated tumor for HSP70-2, PCNA and cell cycle molecules (CDK1, CDK2, CDK4, CDK6, Cyclin A2, Cyclin B1, Cyclin D1, Cyclin E, p21). H&E staining shows cytostructure of the tumor cells. Objective: ×20, Magnification: ×200. *P < 0.05, **P < 0.001. Data are represented as mean ± SEM

To validate our finding on HSP70-2 in cell culture, we studied the effect of ablation of HSP70-2 on tumor growth in xenograft mouse model. As shown in Fig. 5a, there was a reduction in tumor size in HSP70-2 shRNA4 treated mice compared to NC shRNA treated mice. The tumor weight and volume of mice injected with HSP70-2 shRNA4 was significantly reduced (*P < 0.001*) as compared control animals (Fig. 5b, c). Western blot analysis of xenograft tissue sections showed that HSP70-2 protein and PCNA was reduced post HSP70-2 shRNA4 administration (Fig. 5d). Further, IHC analysis of excised tumor sections confirmed reduction in HSP70-2 and PCNA expression (Fig. 5e).

**Fig. 6:**
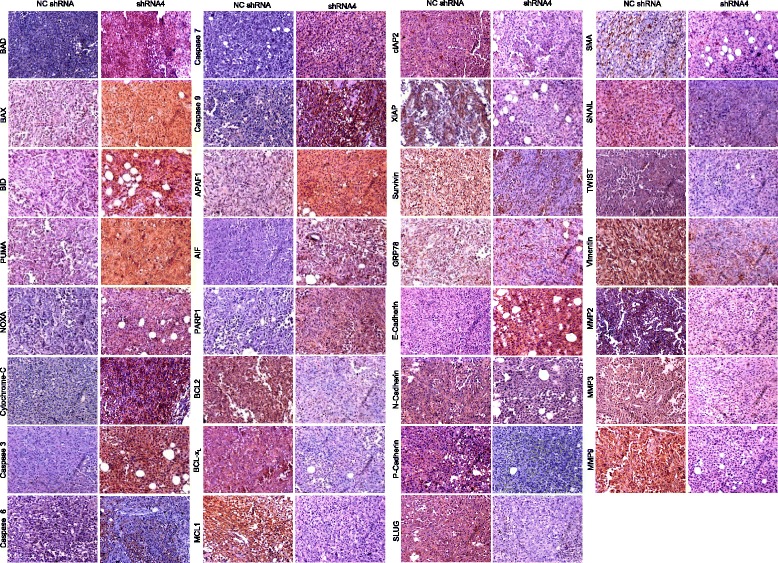
HSP70-2 depletion in MDA-MB-231 tumor cells initiates apoptosis and inhibits EMT in tumor xenograft. Representative IHC images show altered expression of apoptotic molecules (BAD, BAX, BID, PUMA, NOXA, Cytochrome-C, Caspase 3, Caspase 6, Caspase 7, Caspase 9, APAF1, AIF, PARP1, BCL2, BCL-x_L_, MCL1, cIAP2, XIAP, Survivin, GRP78), epithelial (E-Cadherin) and mesenchymal markers (N-Cadherin, P-Cadherin, SLUG, SMA, SNAIL, TWIST, Vimentin, MMP2, MMP3, MMP9) in sections of tumor treated with shRNA4 and NC shRNA. Objective: ×20, Magnification: ×200

We further examined these tumor sections to understand changes in the molecules involved in various pathways following HSP70-2 depletion by IHC. In agreement with our in-vitro data, the IHC analysis showed increased expression of CDK inhibitor, p21 in mice administered with HSP70-2 shRNA4 compared to NC shRNA treated mice (Fig. 5e). This was accompanied with decreased expression of CDKs including CDK1, CDK2, CDK4 and CDK6, cyclins including cyclin A2, cyclin B1, cyclin D1 and cyclin E in tumors treated with HSP70-2 shRNA4 in contrast to NC shRNA. Next, we compared the expression of molecules involved in apoptosis in HSP70-2 shRNA4 and NC shRNA treated tumor (Fig. 6). Notably, most of the key molecules of apoptotic pathway showed increased expression of caspase 3, caspase 6, caspase 7, caspase 9, cytochrome-C, APAF1, BAD, BAX, BID, PUMA and NOXA in HSP70-2 shRNA treated tumors (Fig. 6). This was also concomitant with down-regulation of several anti-apoptotic molecules, BCL2, BCL-x_L_, MCL1, Survivin, cIAP2 and XIAP (Fig. 6). In addition, caspase independent AIF mediated cell death also increased as revealed by increased expression of AIF and PARP1 in HSP70-2 shRNA treated compared to NC shRNA treated tumors (Fig. 6). Also we found, reduction in expression of glucose-regulated protein, GRP78 in tumors treated with HSP70-2 shRNA4 (Fig. 6).

We further investigated the expression of EMT regulators. We observed reduction in the expression of EMT regulator SNAIL, in consequence to the ablation of HSP70-2. In addition, there was a decreased expression of mesenchymal markers such as N-Cadherin, P-Cadherin, Vimentin, SNAIL, SLUG, TWIST and SMA in HSP70-2 shRNA4 treated tumor compared to NC shRNA treated tumors. HSP70-2 ablation also revealed increase in epithelial marker, E-Cadherin expression (Fig. 6). Our data revealed that HSP70-2 shRNA4 treated tumors exhibited decreased expression of MMP2, MMP3 and MMP9 (Fig. 6). To validate our IHC finding, we employed qPCR to check mRNA expression of several key molecules in HSP70-2 shRNA4 treated tumors, relative to the NC shRNA treated tumors. It is interesting to note that our qPCR results were consistent with IHC analysis. Initially we checked the relative level of mRNA of *HSP70-2* in HSP70-2 shRNA4 treated tumors and NC shRNA treated tumors and found a marked depletion of *HSP70-2* mRNA expression upon shRNA mediated gene silencing (Additional file 6: Figure S5a). There was a reduction in *CDK1* (*P* < 0.028), *CDK2* (*P* < 0.039), *CDK, 4* (*P* < 0.001), *CDK6* (*P* < 0.016)*, cyclin B1* (*P* < 0.001)*, cyclin D1* (*P* < 0.001) and *cyclin E* (*P* < 0.001) expression, relative to the NC shRNA treated tumors (Additional file 6: Figure S5b). Our qPCR study on apoptotic molecules in HSP70-2 shRNA4 treated tumors revealed an increase expression of *BAD* (*P* < 0.004)*, caspase 6* (*P* < 0.029)*, caspase 7* (*P* < 0.007)*, caspase 9* (*P* < 0.016)*, PUMA* (*P* < 0.008)*, NOXA* (*P* < 0.001) *and cytochrome-C* (*P* < 0.001). Also decreased expression of anti-apoptotic molecules including *BCL2* (*P* < 0.001), *BCL-x*_*L*_ (*P* < 0.001), *Survivin* (*P* < 0.028), *MCL1* (*P* < 0.005) and *cIAP2* (*P* < 0.001) were found (Additional file 6: Figure S5c). Comparison of mRNA expression of EMT molecules in HSP70-2 shRNA treated tumors and NC shRNA treated tumors revealed decreased expression of *SLUG* (*P* < 0.013)*, P-Cadherin* (*P* < 0.038)*, N-Cadherin* (*P* < 0.001)*, MMP3* (*P* < 0.001)*, MMP9* (*P* < 0.001)*, SMA* (*P* < 0.029) and *SNAIL* (*P* < 0.008). However, *E-Cadherin* (*P* < 0.001), displayed increased expression of 2.55 fold in HSP70-2 shRNA treated mice (Additional file 6: Figure S5d). Interestingly, our IHC data and qPCR results showed similar results.

Collectively, our results indicated that HSP70-2 contributes in cellular proliferation, cellular migration and invasion and its depletion causes significant reduction in these processes both in-vitro and in-vivo xenograft mouse model.

## Discussion

Breast cancer is the most common cancer in women worldwide and is the second leading cause of cancer-related death in women [1]. Among various histotypes of breast cancer, infiltrating ductal carcinoma (IDC) is the most common histotype of breast cancer in which cancer cells invade through the ductal wall into the stroma [2]. Therefore, the present study was undertaken to investigate the role of a novel cancer testis (CT) antigen, HSP70-2 expression and its potential involvement in breast cancer patients and in various breast cancer cell line models. We found that majority of breast cancer patients [83 % (128/154), Table 1] showed HSP70-2 expression irrespective of stages, grades and histotypes. More importantly, we also found significant association of HSP70-2 expression with grades. In addition, HSP70-2 expression was observed in majority of patients with positive lymph node involvement suggesting a role of HSP70-2 in tumor migration and invasion. Our data from various breast cancer cell lines were broadly in agreement with our in-vivo studies in xenograft model. Earlier studies in small number of breast cancer specimens [36 % (9/25)] also revealed HSP70-2 expression [8]. Moreover our recent studies on well characterized CT antigens, SPAG9 and AKAP4 have shown their association with breast cancer cases [14, 15]. The other CT antigens family member, MAGE-A9 and MAGE-A11 have also been found to be expressed in breast cancer specimens [16]. Our current findings in various stages and grades of breast cancer tumors suggest that HSP70-2 may be involved in various signaling pathways and control cellular proliferation, dysregulation of cell cycle, migration and invasion abilities in cancer cells.

Altered expression of cell-cycle-regulatory proteins is a major abnormalities during cancer [17]. It may involve over-expression of cyclins and cyclin dependent kinases (CDK) or mutation of tumor suppressor genes [18]. A earlier report has shown that HSP70-2 is a binding partner of cdk1/cyclinB complex during meiotic division in spermatogenesis [19]. The present study shows that ablation of HSP70-2 resulted in accumulation of cancer cells in G_0_/G_1_ stage. In support of our observation, similar findings have been reported in HSP70-2 depleted HeLa cells [8]. Our results further revealed that molecules involved in cell cycle (cyclins: cyclin A2, cyclin B1, cyclin D1 and cyclin E along with CDKs: CDK1, CDK2, CDK4 and CDK6) were downregulated in HSP70-2 depleted MDA-MB-231 cells. Interestingly, we also found that senescence was associated with G_0_/G_1_ arrest with increased expression of p21, Rb and DCR2 (senescence marker). In this context, it may be possible that the HSP70-2 expression may play an important role in alteration of cell cycle regulation molecules resulting in uncontrolled cellular proliferation.

Failure in process of apoptosis results in cancer disease [17]. The cell death occurs due to initiation of apoptosis which includes well regulated expression of pro- and anti-apoptotic molecules of the Bcl-2 family as regulatory proteins [17]. Over-expression of BCL-2 has been found in 60–80 % of breast carcinoma suggesting its role in breast cancer [20]. The current study showed significant increase in cell death in HSP70-2 depleted MDA-MB-231 and MCF7 cells. Our observations further supported that cancer cell death occurred due to decreased expression in anti-apoptotic molecules (BCL-x_L_, BCL2, Survivin, XIAP2, cIAP2, MCL1) and increased expression in pro-apoptotic molecules (caspase 3, caspase 6, caspase 7, caspase 9, BAX, BAK, BAD, NOXA, BID, cytochrome-C, PUMA, APAF1, AIF, and PARP1) in HSP70-2 depleted MDA-MB-231 cells. Similar findings on BORIS-specific siRNA treatment induced caspase 3/7 activation in a dose dependent manner in MDA-MB-231 cells [21] lead to cell death indicating its role in survival of cancer cells. Interestingly, recent studies have shown the role of yet another important molecule, unfolded protein response (UPR) which is upregulated in triple negative breast cancer (TNBC) [22]. Interestingly, our report on HSP70-2 ablated in TNBC, MDA-MB-231 cells lead to decrease expression of glucose-regulated protein (GRP78), which is a master regulator of UPR and has been associated with cancer disease and drug resistance [23]. Collectively, our study indicates that ablation of HSP70-2 in breast cancer cells promotes apoptosis and hence cancer cell death.

Our most important findings suggest that HSP70-2 expression in breast cancer cells play an important role in cellular growth, cell migration, and invasion. Epithelial-Mesenchymal transition (EMT) is considered to be the important pathway involved in migration and invasion of cancer cells to distant sites [17]. Expression of proteins characteristic of mesenchymal cells (N-Cadherin, Vimentin, SNAIL, SLUG) and loss of epithelial markers (E-Cadherin) correlates with tumor progression and poor prognosis [24]. Our observations supported previous studies [24] wherein HSP70-2 ablation in the present study effected cancer cell migration and invasion potential in MDA-MB-231 and MCF7 cells. Supported by previous findings [24], our data also revealed that various mesenchymal markers like N-Cadherin, P-Cadherin, MMP2, MMP3, MMP9, SLUG, TWIST, Vimentin, and SMA were down-regulated with increased expression of E-Cadherin in HSP70-2 depleted MDA-MB-231 cells. Recent studies on CT antigens such as SSX, MAGED4B, CAGE and piwil2 showed up-regulation of EMT and metastatic genes expression that promote tumor dissemination [25]. A recent study also showed that over-expression of CT45A1, CT antigen in breast cancer cells selectively enhanced the expression of pro-EMT gene, including TWIST1, ALDH1A1 [26]. On similar lines, our data suggests that HSP70-2 depletion in breast cancer cells contributes in reduced cancer cell motility by inhibiting molecules involved in EMT pathway.

In summary, this investigation of well characterized human breast cancer tissues (103 ANCT and *n* = 154 cancer tissue) has documented that HSP70-2 expression is over expressed in breast cancer patients. Furthermore, our data demonstrated that plasmid-mediated RNA interference of HSP70-2 successfully inhibited the expression of HSP70-2 in in-vitro and in-vivo models of breast cancer, leading to inhibitory effects on cell proliferation, migration, invasion, and tumor growth. In this regard, recent, clinical trials employing gene silencing approach has shown a promise in developing new class of therapeutics [27]. Collectively, our findings suggest that the shRNA mediated gene silencing approach may be an effective therapeutic strategy as an adjuvant therapy or in combination with other treatment modalities for breast cancer and therefore warrant future investigations in human clinical trials.

## Conclusions

Taken together, our study indicated that HSP70-2 might be playing an important role in development and progression of breast cancer. Gene silencing approach indicated that HSP70-2 promotes cell growth and cellular motility of breast cancer cells. Also, HSP70-2 depletion led to reduction in tumor growth in in-vivo human breast cancer xenograft model. Recent clinical trials on siRNA based therapy [27] indicated that gene silencing approach has a potential to be employed in cancer therapy. Thus, HSP70-2 may be considered as potential candidate molecule in the management of breast cancer patients and warrants future studies.

## Additional files

Additional file 1: Supplementary Methods, Supplementary table, Supplementary Figure Legends. (DOCX 41 kb) Additional file 2: Figure S1. HSP70-2 expression in breast cancer. (PPTX 1156 kb) Additional file 3: Figure S2. HSP70-2 protein ablation reduces cell viability and colony formation ability of breast cancer cells. (PPTX 203 kb) Additional file 4: Figure S3. HSP70-2 knockdown initiates apoptosis in breast cancer cells. (PPTX 2620 kb) Additional file 5: Figure S4. Depletion of HSP70-2 in breast cancer cells inhibits cellular motility. (PPTX 274 kb) Additional file 6: Figure S5. Quantitative PCR analysis of various genes involved in different signaling cascades in breast cancer tumor xenograft. (PPTX 93 kb)

## References

[1] Siegel R, Miller KD, Jemal A (2016). Cancer statistics, 2016. CA Cancer J Clin.

[2] Makki J (2015). Diversity of Breast Carcinoma: Histological Subtypes and Clinical Relevance. Clin Med Insights Pathol.

[3] Yoder BJ, Wilkinson EJ, Massoll NA (2007). Molecular and morphologic distinctions between infiltrating ductal and lobular carcinoma of the breast. Breast J.

[4] Vineis P, Wild CP (2014). Global cancer patterns: causes and prevention. Lancet.

[5] Calderwood SK, Khaleque MA, Sawyer DB, Ciocca DR (2006). Heat Shock proteins in cancer: chaperones of tumorigenesis. Trends Biochem Sci.

[6] Straume O, Shimamura T, Lampa MJ, Carretero J, Øyan AM, Jia D (2012). Suppression of heat shock protein 27 induces long-term dormancy in human breast cancer. Proc Natl Acad Sci U S A.

[7] Cheng Q, Chang JT, Geradts J, Neckers LM, Haystead T, Spector NL (2012). Amplification and high-level expression of heat shock protein 90 marks aggressive phenotypes of human epidermal growth factor receptor 2 negative breast cancer. Breast Cancer Res.

[8] Rohde M, Daugaard M, Jensen MH, Helin K, Nylandsted J, Jäättelä M (2005). Members of the heat-shock protein 70 family promote cancer cell growth by distinct mechanisms. Genes Dev.

[9] Garg M, Kanojia D, Seth A, Kumar R, Gupta A, Surolia A (2010). Heat-shock protein 70-2 (HSP70-2) expression in bladder urothelial carcinoma is associated with tumor progression and promotes migration and invasion. Eur J Cancer.

[10] Garg M, Kanojia D, Saini S, Suri S, Gupta A, Surolia A (2010). Germ cell-specific heat shock protein 70-2 is expressed in cervical carcinoma and is involved in the growth, migration and invasion of cervical cells. Cancer.

[11] Singh S, Suri A (2014). Targeting the testis-specific heat-shock protein 70-2 (HSP70-2) reduces cellular growth, migration, and invasion in renal cell carcinoma cells. Tumor Biol.

[12] Kanojia D, Garg M, Gupta S, Gupta A, Suri A (2009). Sperm-Associated Antigen 9, a Novel Biomarker for Early Detection of Breast Cancer. Cancer Epidemiol Biomarkers Prev.

[13] Kanojia D, Garg M, Saini S, Agarwal S, Parashar D, Jagadish N (2013). Sperm associated antigen 9 plays an important role in bladder transitional cell carcinoma. PLoS One.

[14] Jagadish N, Parashar D, Gupta N, Agarwal S, Purohit S, Kumar V (2015). A-kinase anchor protein 4 (AKAP4) a promising therapeutic target of colorectal cancer. J Exp Clin Cancer Res.

[15] Saini S, Jagadish N, Gupta A, Bhatnagar A, Suri A (2013). A novel cancer testis antigen, a-kinase anchor protein 4 (AKAP4) is a potential biomarker for breast cancer. PLoS ONE.

[16] Hou SY, Sang MX, Geng CZ, Liu WH, Lü WH, Xu YY (2014). Expressions of MAGE-A9 and MAGE-A11 in breast cancer and their expression mechanism. Arch Med Res.

[17] Hanahan D, Weinberg RA (2011). Review Hallmarks of Cancer : The Next Generation. Cell.

[18] Williams GH, Stoeber K (2012). The cell cycle and cancer. J Pathol.

[19] Zhu D, Dix DJ, Eddy EM (1997). HSP70-2 is required for CDC2 kinase activity in meiosis I of mouse spermatocytes. Development.

[20] Lindeman GJ, Visvader JE (2013). Targeting BCL-2 in breast cancer: exploiting a tumor lifeline to deliver a mortal blow?. Breast Cancer Manag.

[21] Dougherty CJ, Ichim TE, Liu L, Reznik G, Min WP, Ghochikyan A (2008). Selective apoptosis of breast cancer cells by siRNA targeting of BORIS. Biochem Biophys Res Commun.

[22] Chen X, Iliopoulos D, Zhang Q, Tang Q, Greenblatt MB, Hatziapostolou M (2014). XBP1 promotes triple-negative breast cancer by controlling the HIF1α pathway. Nature.

[23] Wang J, Yin Y, Hua H, Li M, Luo T, Xu L (2009). Blockade of GRP78 sensitizes breast cancer cells to microtubules-interfering agents that induce the unfolded protein response. J Cell Mol Med.

[24] Thiery JP, Sleeman JP (2006). Complex networks orchestrate epithelial-mesenchymal transitions. Nat Rev Mol Cell Biol.

[25] Yang P (2015). Cancer/Testis Antigens Trigger Epithelial-Mesenchymal Transition and Genesis of Cancer Stem-Like Cells. Curr Pharm Des.

[26] Shang B, Gao A, Pan Y, Zhang G, Tu J, Zhou Y (2014). CT45A1 acts as a new proto-oncogene to trigger tumorigenesis and cancer metastasis. Cell Death Dis.

[27] Tabernero J, Shapiro GI, LoRusso PM, Cervantes A, Schwartz GK, Weiss GJ (2013). First-in Humans Trial of an RNA Interference Therapeutic Targeting VEGF and KSP in Cancer Patients with Liver Involvement. Cancer Discov.

