# Supplementary material for: Heat shock protein 70-2 (HSP70-2) overexpression in breast cancer
Source: J Exp Clin Cancer Res. 2016 Sep 22;35:150. doi: 10.1186/s13046-016-0425-9 (PMC5034467; doi:10.1186/s13046-016-0425-9)
Supplement: Additional file 1: — Supplementary Methods, Supplementary table, Supplementary Figure Legends. (DOCX 41 kb) [file 13046_2016_425_MOESM1_ESM.docx]

**Supplementary Information**

**1. Supplementary Methods**

**2. Supplementary table**

**3. Supplementary Figure Legends**

**1. Supplementary Methods**

**RT-PCR and Real-time PCR**

Total RNA was isolated from the patient tumor specimens, breast cancer cell lines and Human normal mammary epithelial cells (HNMEC's) using cells using RNeasy mini kit (Qiagen, Germany) as per manufacturer's instruction. *In-vitro* synthesis of cDNA was done using High Capacity cDNA Reverse Transcription Kits (Applied Biosystems, USA) from 500ng of total RNA. Subsequently, RT-PCR was carried out from cDNA template using HSP70-2 specific primers (Forward primer: 5’-CCACAGTGCAGTCGGATATG-3’; Reverse primer 5’-GCGATCTCCTTCATCTTCGT-3’). *β-actin* was used as an internal control in all the reactions (Forward primer 5’-ATCTGGCACCACACCTTCTACAATGAGCTGCG-3′; Reverse primer 5’-CGTCATACTCCTGCTTGCTGATCCACATCTGC-3′). The products were cloned into TOPO vector (Invitrogen, Carlsbad, CA) to confirm the sequence of the amplicon. In addition, real-time PCR was also carried out to check the expression levels of HSP70-2 mRNA in the breast cancer cell lines compared to HNMEC's as described earlier [1]. Reaction was done using 10ng of cDNA from HNMEC's and breast cancer cell lines with SYBR Green Real time PCR master mix (Bio-Rad, CA, USA) on an iCycler iQ multicolor real-time PCR detection system (Bio-Rad, CA, USA). *HSP70-2* gene expression levels in each sample were subsequently normalized using expression level of *β-actin* in the same mRNA sample as endogenous gene.

**Western blotting**

HSP70-2 protein expression in breast cancer cells was checked by Western blotting using cell lysates of various cancer cells. Cell lysates were resolved on sodium dodecylsulphate-polyacrylamide gel electrophoresis (SDS-PAGE). To detect the presence of HSP70-2 protein, rabbit anti-HSP70-2 antibody was used as primary antibody and goat anti-rabbit IgG Horseradish Peroxidase (HRP) (Jackson Immuno Research Laboratories, Inc., Baltimore, USA) as secondary antibody. Immunoreactivity against HSP70-2 protein was developed by Immobilon Western Chemiluminescent HRP substrate (Millipore Corporation, USA).

**Flow cytometry**

Surface localization of HSP70-2 was detected in all breast cancer cell lines using flow-cytometric analyses as described previously [2]. Cells were first fixed with 0.4% paraformaldehyde. Cells were then incubated with anti-HSP70-2 antibody or control IgG followed by goat anti-rabbit IgG fluorescein isothiocyanate conjugate (FITC, Jackson Immuno Research Laboratories, Inc., Baltimore, USA) as secondary antibody. The cells were acquired and analyzed on BD-CALIBUR (BD Biosciences, California, USA). The three independent experiments were carried out in triplicates.

**Indirect Immunofluoroscence**

Cellular localization of HSP70-2 protein was demonstrated in all breast cancer cell lines using anti-HSP70-2 antibody or a control IgG by immunofluorescence assay as described previously [2]. Cells were fixed with 3% paraformaldehyde and permeabilized with 0.5% IGEPAL. Subsequently cells were blocked with 5% normal donkey sera. Blocked cells were incubated with anti-HSP70-2 antibody followed by goat anti-rabbit IgG FITC (Jackson Immuno Research Laboratories, Inc., Baltimore, USA) as secondary antibody. Nuclear staining of cells was done using 4,6-diamidino-2-phenylindole (DAPI, Sigma-Aldrich, St. Louis, MO, USA). The slides were mounted in antifade reagent (Invitrogen Life Technologies Corporation, USA). The photomicrographs were captured using Carl Zeiss LSM 510 Meta confocal microscope (Germany).

In addition, to study the co-localization of HSP70-2 with various sub-cellular organelles, all the four breast cancer cells were also probed with antibodies to endoplasmic reticulum (calnexin, Santa Cruz Biotechnology, Santa Cruz, CA), Golgi bodies (GM130 Santa Cruz Biotechnology), mitochondria (MTCO2, Abcam, Cambridge, United Kingdom) and nuclear envelope (lamin A/C Santa Cruz Biotechnology). Texas red conjugated anti-mouse IgG was used as secondary antibody for co-localization. Photo micrographs were captured using the Carl Zeiss LSM 510 Meta confocal microscope (Germany) in central confocal microscopy facility.

**Immunohistochemistry**

The cellular localization of HSP70-2 protein was visualized in all the breast cancer tissue specimens as described earlier [2]. Briefly, paraffin-embedded tissue sections were blocked with normal goat serum after deparaffinization and rehydration. Specimens were incubated with anti-HSP70-2 antibody or control IgG followed by incubation with goat anti-rabbit IgG HRP (Jackson Immuno Research Laboratories, Inc., Baltimore, USA) as secondary antibody. The immuno-reactivity was visualized using 3,3'-diaminobenzidine (DAB, Sigma-Aldrich, St. Louis, MA). The images were captured using Nikon Eclipse E400 microscope (Nikon, Fukok, Japan).

**Validation of shRNA target**

The following target sequences were used: shRNA1: 5’- CATAACGGTCCCGGCCTATT-3’; shRNA2: 5’-GAGCGGTACAAATCGGAAGAT-3’; shRNA3:5’-CGGCGACAAATCAGAGAATGT-3’; shRNA4: 5’-TCGACGCCAAGAGGCTGATT-3’ and NC shRNA: 5’-GGAATCTCATTCGATGCATAC-3’ were used for gene silencing studies. The transient transfections were carried out in MCF7 and MDA-MB-231 cell lines using lipofectamine (Invitrogen Life Technologies Corporation, USA). After 48h cells were harvested to prepare mRNA and cell lysate for protein estimation. Total RNA was extracted from NC shRNA and HSP70-2 shRNA (all four targets) transfected cells using RNeasy mini kit (Qiagen, Germany). Complimentary DNA (cDNA) was synthesized using High Capacity cDNA Reverse Transcription Kits (Applied Biosystems, USA) and real time-PCR was performed using HSP70-2 primers. Also, real-time PCR was carried out for different molecules involved in apoptosis, cell cycle and EMT using specific primers as listed in supplementary table 1.

In addition, efficiency of shRNA targets against HSP70-2 was also determined by Western blot analysis as described earlier [2] using rabbit anti-HSP70-2 antibody and HRP conjugated anti-rabbit IgG secondary antibody raised in goat (Jackson Immuno Research Laboratories, Inc., Baltimore, USA). Immunoreactivity against HSP70-2 protein was developed by Immobilon Western Chemiluminescent HRP substrate (Millipore Corporation, USA). The two shRNA targets that showed maximum knockdown efficiency were further used for the *in-vitro* and *in-vivo* assays. In addition, Western blotting was also done for different molecules involved in apoptosis, cell cycle and EMT as detailed in supplementary table 2.

**Cellular proliferation, cell viability and colony forming ability**

To study the cellular proliferation, HSP70-2 shRNA3, shRNA4 and NC shRNA transfected MCF7 and MDA-MB-231 cells were counted at 24h, 48h and 72h after seeding the cells. Cell viability assay was performed as described earlier [2]. HSP70-2 shRNA3, shRNA4 and NC shRNA transfected MCF7 and MDA-MB-231 cells were treated with MTT reagent and absorbance taken at 570nm on ELISA reader (BioTek μQuant Microplate Spectrophotometer) using KC junior software. Colony formation assay of HSP70-2 shRNA3, shRNA4 and NC shRNA transfected MCF7 and MDA-MB-231 cells was carried out as described earlier [2]. Cells were seeded at low density (400-1200 cells per well) and number of colonies were counted after ten days. The three independent experiments were carried out in triplicates.

**Migration, Invasion and wound healing assay**

For migration assay, HSP70-2 shRNA3, shRNA4 and NC shRNA transfected MCF7 and MDA-MB-231 cells (1x10^5^ cells per insert) were seeded in serum free media onto 8-μm pore inserts with serum containing media in lower chamber. For invasion assay, inserts were coated with 60µl of 5mg/ml matrigel (BD Biosciences, California, USA) and the assay was carried out similarly. The migrating and invading cells were stained after 48h and counted. For wound healing assay, wound was mechanically created on a confluent monolayer using sterile 200μl pipette tip. Images were taken at regular interval.The three independent experiments were carried out in triplicates.

***In-vivo* xenograft studies**

Athymic nude mice (National Institute of Immunology[NII], National Institutes of Health, [S] nu/nu) were utilized after obtainingapproval from animal ethical committee of NII for the xenograft studies. Mice were injected subcutaneously with 5x10^6^ MDA-MB-231 cells. When the tumor size of 50-100 mm^3^ was achieved, the mice were divided in two groups of 8 mice each: control and experimental. Intra-tumor injections (first booster dose of 50µg followed by maintenance dose of 25µg) of NC shRNA and HSP70-2 shRNA4 were administered thrice weekly to the control and experimental groups respectively for 49 days. Tumor volume was monitored daily. After 49 days tumors were excised for further experiments. IHC was performed to check HSP70-2 and PCNA expression in HSP70-2 shRNA4 and NC shRNA treated tumors. Further, the serial sections were also probed for various molecules involved in apoptosis, EMT and cell cycle. Tumor lysates were also checked for HSP70-2 protein and PCNA. A portion of tumor was also taken in RNA*later* to carry out gene expression studies.

**References**

1. Sinha A, Agarwal S, Parashar D, Verma A, Saini S, Jagadish N, et al. Down regulation of SPAG9 reduces growth and invasive potential of triple-negative breast cancer cells: possible implications in targeted therapy. J Exp Clin Cancer Res. 2013;32:69.
2. Garg M, Kanojia D, Saini S, Suri S, Gupta A, Surolia A, et al. Germ cell-specific heat shock protein 70-2 is expressed in cervical carcinoma and is involved in the growth, migration, and invasion of cervical cells. Cancer. 2010;116:3785-3796.

**2. Supplementary tables**

**Supplementary table S1: Primers**

| **S.No** | **Gene** | **Primer Sequence (5’-3’)** |
| --- | --- | --- |
|  | **Apoptosis studies** | |
| 1 | *Cytochrome-c* | Forward: GGCGTGTCCTTGGACTTAGA  Reverse: TGCCTTTCTCAACATCACCC |
| 2 | *Caspase 6* | Forward: AAGAGGAGGGCAAGGTGTCT  Reverse: TTTCTGTCATGTTTTCTTCCCC |
| 3 | *Caspase 7* | Forward: GGTTGAGGATTCAGCAAATGA  Reverse: GGATCGCATGGTGACATTTT |
| 4 | *Caspase 9* | Forward: AGGTTCTCAGACCGGAAACA  Reverse: CTGCATTTCCCCTCAAACTC |
| 5 | *BCL2* | Forward: CTGAGTACCTGAACCGGCA  Reverse: GAGAAATCAAACAGAGGCCG |
| 6 | *BCL-x_L_* | Forward: TTCAGTGACCTGACATCCCA  Reverse: CTGCTGCATTGTTCCCATAG |
| 7 | *MCL1* | Forward: TCGTAAGGACAAAACGGGAC  Reverse: CATTCCTGATGCCACCTTCT |
| 8 | *Survivin* | Forward: TTGGTGAATTTTTGAAACTGGA  Reverse: CTTTCTCCGCAGTTTCCTCA |
| 9 | *cIAP2* | Forward: GTCAAATGTTGAAAAAGTGCCA  Reverse: GGGAAGAGGAGAGAGAAAGAGC |
| 10 | *BAD* | Forward: CAGGCCTCCTGTGGGAC  Reverse: GGTAGGAGCTGTGGCGACT |
| 11 | *PUMA* | Forward: GACGACCTCAACGCACAGTA  Reverse: GTAAGGGCAGGAGTCCCAT |
| 12 | *NOXA* | Forward: AAGTTTCTGCCGGAAGTTCA  Reverse: GCAAGAACGCTCAACCGAG |
| 13 | *XIAP* | Forward: GACCCTCCCCTTGGACC  Reverse: CTGTTAAAAGTCATCTTCTCTTGAAA |

|  | **Cell cycle studies** | |
| --- | --- | --- |
| 1 | *Cyclin B1* | Forward: CAGATGTTTCCATTGGGCTT  Reverse: GAACCTGAGCCAGAACCTGA |
| 2 | *Cyclin D1* | Forward: GGCGGATTGGAAATGAACTT  Reverse: TCCTCTCCAAAATGCCAGAG |
| 3 | *Cyclin E* | Forward: TCTTTGTCAGGTGTGGGGA  Reverse: GAAATGGCCAAAATCGACAG |
| 4 | *CDK1* | Forward: GCGGAATAATAAGCCGGGAT  Reverse: CAACTCCATAGGTACCTTCTCCA |
| 5 | *CDK2* | Forward: GAATCTCCAGGGAATAGGGC  Reverse: CTGAAATCCTCCTGGGCTG |
| 6 | *CDK4* | Forward: TGCAGTCCACATATGCAACA  Reverse: GTCGGCTTCAGAGTTTCCAC |
| 7 | *CDK6* | Forward: TGTCTGTTCGTGACACTGTGC  Reverse: ATGCCGCTCTCCACCAT |
| 8 | *p21* | Forward: AGTCAGTTCCTTGTGGAGCC  Reverse: CATGGGTTCTGACGGACAT |

|  | **EMT studies** | |
| --- | --- | --- |
| 1 | *E-Cadherin* | Forward: GACCGGTGCAATCTTCAAA  Reverse: TTGACGCCGAGAGCTACAC |
| 2 | *N-Cadherin* | Forward: CCACCTTAAAATCTGCAGGC  Reverse: GTGCATGAAGGACAGCCTCT |
| 3 | *P-Cadherin* | Forward: AGGCTGAAGTGACCTTGGAG  Reverse: CAGTGCTAAACAGAGCTGGC |
| 4 | *SNAIL* | Forward: AGGTTGGAGCGGTCAGC  Reverse: CCTTCTCTAGGCCCTGGCT |
| 5 | *SLUG* | Forward: TGACCTGTCTGCAAATGCTC  Reverse: CAGACCCTGGTTGCTTCAA |
| 6 | *VIMENTIN* | Forward: ATTCCACTTTGCGTTCAAGG  Reverse: CTTCAGAGAGAGGAAGCCGA |
| 7 | *SMA* | Forward: CCAGAGCCATTGTCACACAC  Reverse: CAGCCAAGCACTGTCAGG |
| 8 | *MMP2* | Forward: ATGCCGCCTTTAACTGGAG  Reverse: GGAAAGCCAGGATCCATTTT |
| 9 | *MMP3* | Forward: AGGGATTAATGGAGATGCCC  Reverse: CAATTTCATGAGCAGCAACG |
| 10 | *MMP9* | Forward: ACGACGTCTTCCAGTACCGA  Reverse: TTGGTCCACCTGGTTCAACT |

**Supplementary Table S2: Antibodies for various molecules involved in various signaling pathways**

| **S.No** | **Antibody** | **Catalog number** | **Source** |
| --- | --- | --- | --- |
|  | **Apoptosis studies** | | |
| 1 | Cytochrome-C | sc-13560 | Santa Cruz Biotechnology, Inc., USA |
| 2 | Caspase 3 | sc-56052 | Santa Cruz Biotechnology, Inc., USA |
| 3 | Caspase 6 | ab155241 | Abcam, Cambridge, United Kingdom |
| 4 | Caspase 7 | sc-81654 | Santa Cruz Biotechnology, Inc., USA |
| 5 | Caspase 9 | sc-56077 | Santa Cruz Biotechnology, Inc., USA |
| 6 | PARP1 | CST9532 | Cell Signaling Technology, Danvers, Massachusetts |
| 7 | APAF1 | ab2000 | Abcam, Cambridge, United Kingdom |
| 8 | BCL2 | B3170 | Sigma-Aldrich, St. Louis, MO, USA |
| 9 | BCL-x_L_ | B9429 | Sigma-Aldrich, St. Louis, MO, USA |
| 10 | MCL1 | ab32087 | Abcam, Cambridge, United Kingdom |
| 11 | Survivin | ab24479 | Abcam, Cambridge, United Kingdom |
| 12 | XIAP | ab2541 | Abcam, Cambridge, United Kingdom |
| 13 | cIAP2 | sc-7944 | Santa Cruz Biotechnology, Inc., USA |
| 14 | BAX | B8554 | Sigma-Aldrich, St. Louis, MO, USA |
| 15 | BAK | sc-7873 | Santa Cruz Biotechnology, Inc., USA |
| 16 | BAD | sc-8044 | Santa Cruz Biotechnology, Inc., USA |
| 17 | BID | sc-11423 | Santa Cruz Biotechnology, Inc., USA |
| 18 | PUMA | ab54288 | Santa Cruz Biotechnology, Inc., USA |
| 19 | NOXA | sc-56169 | Santa Cruz Biotechnology, Inc., USA |
| 20 | AIF | Ab89583 | Abcam, Cambridge, United Kingdom |
| 21 | GRP78 | Sc-166490 | Santa Cruz Biotechnology, Inc., USA |

|  | **Cell cycle and Senescence studies** | | |
| --- | --- | --- | --- |
| 1 | Cyclin A2 | ab137769 | Abcam, Cambridge, United Kingdom |
| 2 | Cyclin B1 | sc-7393 | Santa Cruz Biotechnology, Inc., USA |
| 3 | Cyclin D1 | sc-8396 | Santa Cruz Biotechnology, Inc., USA |
| 4 | Cyclin E | sc-56310 | Santa Cruz Biotechnology, Inc., USA |
| 5 | p21 | sc-817 | Santa Cruz Biotechnology, Inc., USA |
| 6 | CDK1 | ab18 | Abcam, Cambridge, United Kingdom |
| 7 | CDK2 | ab7954 | Abcam, Cambridge, United Kingdom |
| 8 | CDK4 | sc-23896 | Santa Cruz Biotechnology, Inc., USA |
| 9 | CDK6 | sc-7961 | Santa Cruz Biotechnology, Inc., USA |
| 10 | Rb | ab24 | Abcam, Cambridge, United Kingdom |
| 11 | p-Rb | ab76298 | Abcam, Cambridge, United Kingdom |
| 12 | DcR2 | ab2019 | Abcam, Cambridge, United Kingdom |
| 13 | Lamin B1 | ab16048 | Abcam, Cambridge, United Kingdom |

|  | **EMT studies** | | |
| --- | --- | --- | --- |
| 1 | E-Cadherin | ab1416 | Abcam, Cambridge, United Kingdom |
| 2 | N-Cadherin | ab76011 | Abcam, Cambridge, United Kingdom |
| 3 | P-Cadherin | ab19350 | Abcam, Cambridge, United Kingdom |
| 4 | SNAIL | ab85931 | Abcam, Cambridge, United Kingdom |
| 5 | SLUG | ab51772 | Abcam, Cambridge, United Kingdom |
| 6 | VIMENTIN | ab92547 | Abcam, Cambridge, United Kingdom |
| 7 | SMA | ab7817 | Abcam, Cambridge, United Kingdom |
| 8 | MMP2 | ab92536 | Abcam, Cambridge, United Kingdom |
| 9 | MMP3 | ab52915 | Abcam, Cambridge, United Kingdom |
| 10 | MMP9 | ab119906 | Abcam, Cambridge, United Kingdom |
| 11 | TWIST | ab50887 | Abcam, Cambridge, United Kingdom |

**3. Supplementary Figure Legends**

**Figure S1**. HSP70-2 expression in breast cancer. **a.** Representative images in first panel shows H&E staining in different histotypes of breast cancer. Second panel shows no reactivity in tissue sections when probed with control IgG antibody. Third panel shows no immuno-reactivity in ANCT specimens when probed with anti-HSP70-2 antibody. **b.** First panel shows representative images for H&E staining of serial sections of different stages of IDC histotype. Second panel shows no reactivity in sections stained with control IgG antibody. Third panel shows no reactivity in ANCT specimens stained with anti-HSP70-2 antibody. **c.** First panel shows cytostructure in different grades of breast cancer specimens by H&E staining. Second panel shows depicts increasing PCNA immuno-reactivity (chocolate brown) in grade 1 to grade 3. Third panel shows no immuno-reactivity in tissue specimens when stained with control IgG.DCIS: Ductal Carcinoma *in-situ*, IDC: Infiltrating Ductal carcinoma and ILC: Infiltrating Lobular Carcinoma. Objective: x20, Magnification: x200. **d.** Bar diagrams show HSP70-2 immuno-reactivity Score (IRS) score comparison among Group I and Group II, early and late stage, stage I-IV of IDC histotype, Grades 1-3 of IDC histotype, histotypes of breast cancer, lymph node involvement. **P<0.05, **P<0.0001*. **e.** IIF images show cytoplasmic localization (green) of HSP70-2 in BT-474, MCF7, MDA-MB-231 and SK-BR-3 breast cancer cells. Nucleus was stained with DAPI. Objective: x63, Magnification: x630.

**Figure S2**. HSP70-2 protein ablation reduces cell viability and colony formation ability of breast cancer cells. **a.** Histogram shows significant difference in cell viability of MCF7 and MDA-MB-231 cells transfected with shRNA3, shRNA4 compared to NC shRNA post 24h, 48h and 72h. **b.** Histogram demonstrates significant reduction in colony forming ability of MCF7 and MDA-MB-231 cells transfected with shRNA3, shRNA4 compared to NC shRNA. The three independent experiments were carried out in triplicates.**P<*0.05, ***P<*0.001, ****P<*0.0001. Data are represented as mean±SEM. **c.** FACS analysis depicts accumulation of HSP70-2 depleted MDA-MB-231 cells in G_0_/G_1_ stage of cell cycle as compared to NC shRNA transfected cells. P1: G_0_/G_1_, P2: S, P3: G_2_/M phase. **d.** Histogram depicts qPCR analysis showing reduced relative mRNA expression of CDKs (*CDK1, CDK2, CDK4, CDK6*), cyclins (*Cyclin B1, Cyclin D1, Cyclin E*) and increased expression of p21 in MDA-MB-231 cells transfected with shRNA3, shRNA4 compared to NC shRNA. The three independent experiments were carried out in triplicates.**P<*0.05, ***P<*0.001, ****P<*0.0001. Data are represented as mean±SEM. The three independent experiments were carried out in triplicates.

**Figure S3**. HSP70-2 knockdown initiates apoptosis in breast cancer cells. **a.** Scanning electron microscopy images show no change in phenotypic characteristics of MCF7 and MDA-MB-231 cells treated with lipofectamine (negative control) while, membrane blebbing was observed when the cells were treated with DMSO (positive control). **b.** Histogram depicts qPCR analysis showing up-regulation of pro-apoptotic molecules, *caspase 6, caspase 7, caspase 9, PUMA, cytochrome-C* and down-regulation of anti-apoptotic molecules, *BCL2, BCL-x_L_, Survivin, cIAP2, XIAP, MCL1* in HSP70-2 ablated MDA-MB-231 cells. The three independent experiments were carried out in triplicates.**P<*0.05, ***P<*0.001, ****P<*0.0001. Data are represented as mean±SEM. The three independent experiments were carried out in triplicates. Magnification: x2500, WD=6mm, EHT=20.00kV

**Figure S4**. Depletion of HSP70-2 in breast cancer cells inhibits cellular motility**. a.** Microscopy images show delayed wound healing post 12h, 48h in HSP70-2 depleted MCF7 and MDA-MB-231 cells. Original magnification x100, objective x10. **b.** Histogram shows qPCR analysis of EMT molecules *(P-Cadherin, E-Cadherin, N-Cadherin, MMP2, MMP3, SLUG, SNAIL, Vimentin, SMA*) in MDA-MB-231 cells transfected with shRNA4 as compared to NC shRNA. The three independent experiments were carried out in triplicates. **P<*0.05, ***P<*0.001, ****P<*0.0001. Data are represented as mean±SEM.

**Figure S5.** Quantitative PCR analysis of various genes involved in different signaling cascades in breast cancer tumor xenograft. **a.** Histograms depicts qPCR analysis of *HSP70-2* in shRNA4 treated mice compared to NC shRNA treated mice. **b.** Histogram shows down-regulation of CDKs (*CDK1, CDK2, CDK4, CDK6*) and cyclins (*Cyclin B1, Cyclin D1, Cyclin E*) in shRNA4 treated tumor compared to NC shRNA. **c.** Histogram depicts up-regulation of *Caspase 6, caspase 7, caspase 9, BAD, PUMA, NOXA, cytochrome-C* and down-regulation of *BCL2, BCL-x_L_, MCL1, Survivin, cIAP2* in shRNA4 and NC shRNA treated tumor. **d.** Histogram shows down-regulation of mesenchymal markers (*P-Cadherin, N-Cadherin, MMP3, MMP9, SLUG, SNAIL, SMA*) and up-regulation of epithelial marker (*E-Cadherin*) in tumor cells shRNA4 treated tumor compared to NC shRNA. The three independent experiments were carried out in triplicates. **P<*0.05, ***P<*0.001, ****P<*0.0001. Data are represented as mean±SEM.
