# Supplementary material for: Heat shock protein 70-2 (HSP70-2) overexpression in breast cancer
Source: J Exp Clin Cancer Res. 2016 Sep 22;35:150. doi: 10.1186/s13046-016-0425-9 (PMC5034467; doi:10.1186/s13046-016-0425-9)
Supplement: Additional file 2: Figure S1. — HSP70-2 expression in breast cancer. (PPTX 1156 kb) [file 13046_2016_425_MOESM2_ESM.pptx]

## Slide 1
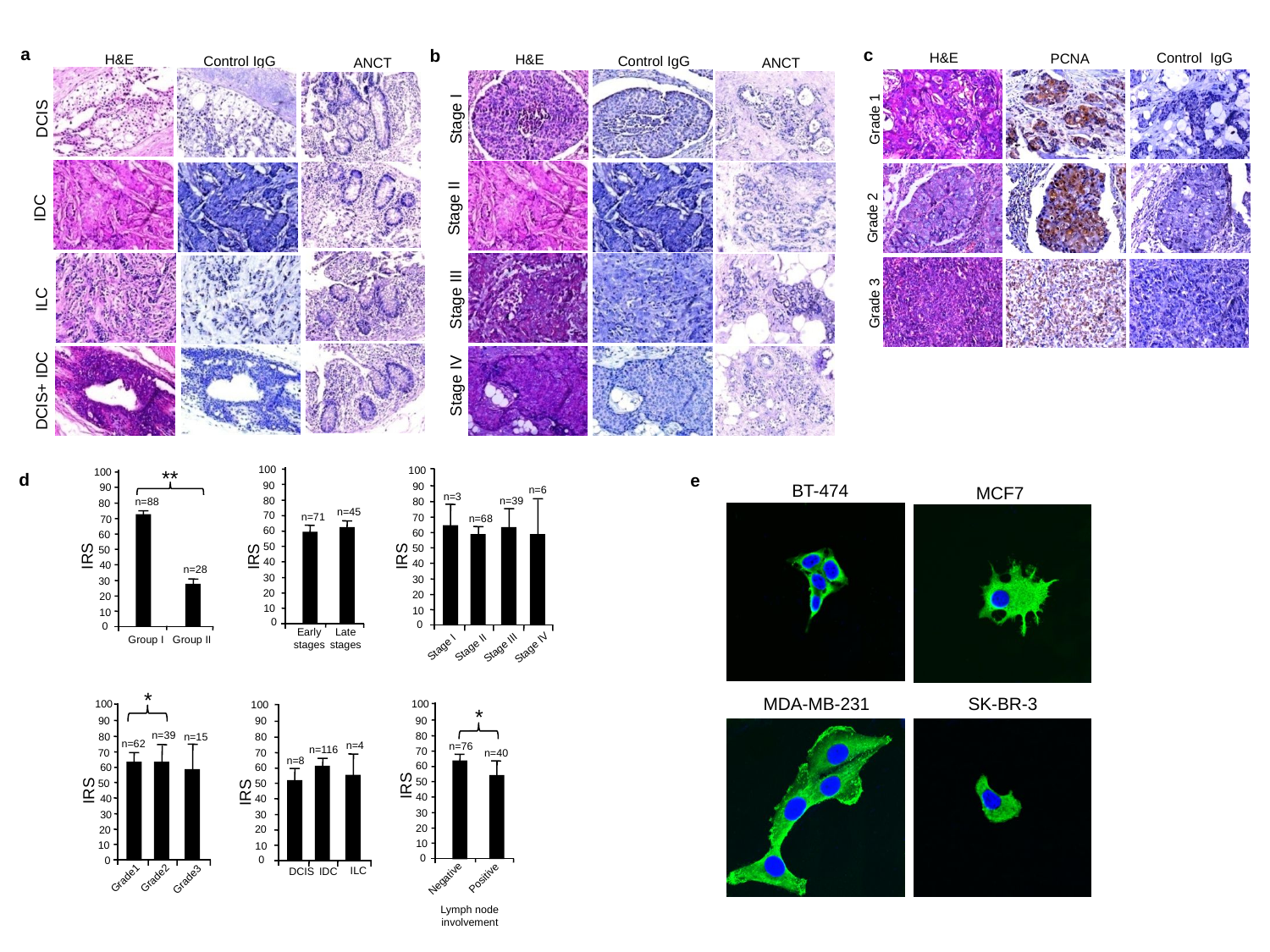

a
c
b
H&E
Control IgG
PCNA
Grade 1
Grade 2
Grade 3
H&E
Control IgG
ANCT
DCIS
IDC
ILC
DCIS+ IDC
H&E
Control IgG
ANCT
Stage I
Stage II
Stage III
Stage IV
100
Late
stages
90
80
70
60
50
40
30
20
10
n=45
n=71
IRS
0
Early
stages
100
90
80
70
60
50
40
30
20
10
Stage I
Stage II
Stage III
Stage IV
n=6
n=3
n=39
n=68
IRS
0
**
100
90
80
70
60
50
40
30
20
10
Group I
n=88
n=28
IRS
0
Group II
*
100
90
80
70
60
50
40
30
20
10
Grade1
Grade2
Grade3
n=39
n=15
n=62
IRS
0
100
90
80
70
60
50
40
30
20
10
n=76
n=40
IRS
0
*
Positive
Negative
Lymph node involvement
100
90
80
70
60
50
40
30
20
10
DCIS
IDC
ILC
n=4
n=116
n=8
IRS
0
d
e
BT-474
MCF7
 SK-BR-3
MDA-MB-231
