# Supplementary material for: Heat shock protein 70-2 (HSP70-2) overexpression in breast cancer
Source: J Exp Clin Cancer Res. 2016 Sep 22;35:150. doi: 10.1186/s13046-016-0425-9 (PMC5034467; doi:10.1186/s13046-016-0425-9)
Supplement: Additional file 3: Figure S2. — HSP70-2 protein ablation reduces cell viability and colony formation ability of breast cancer cells. (PPTX 203 kb) [file 13046_2016_425_MOESM3_ESM.pptx]

## Slide 1
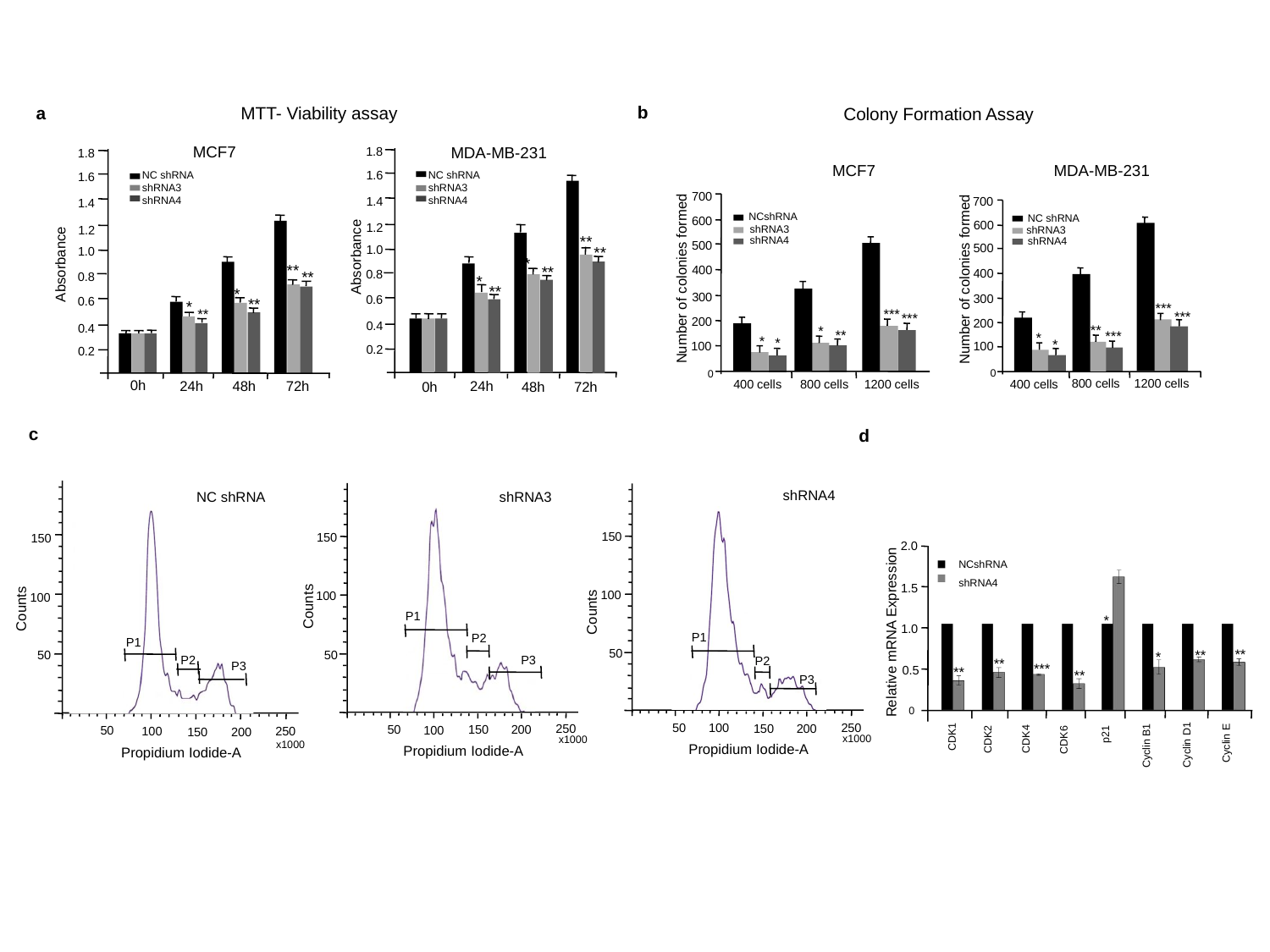

b
a
MTT- Viability assay
Colony Formation Assay
MCF7
1.8
NC shRNA
shRNA3
shRNA4
1.6
1.4
1.2
1.0
Absorbance
**
*
**
48h
**
0.8
0.6
*
**
24h
0.4
0h
0.2
72h
MDA-MB-231
1.8
1.6
1.4
1.2
1.0
0.8
0.6
0.4
0.2
NC shRNA
shRNA3
shRNA4
**
**
*
**
*
**
Absorbance
24h
72h
48h
0h
MCF7
700
600
500
400
300
200
100
0
1200 cells
400 cells
800 cells
Number of colonies formed
***
NCshRNA
 shRNA3
 shRNA4
***
*
**
*
*
MDA-MB-231
700
600
500
400
300
200
100
0
NC shRNA
shRNA3
shRNA4
Number of colonies formed
1200 cells
800 cells
400 cells
***
***
**
***
*
*
c
d
shRNA4
Counts
50
100
250
150
200
Propidium Iodide-A
150
100
50
P1
P2
P3
x1000
Counts
50
100
250
150
200
Propidium Iodide-A
150
100
50
NC shRNA
P1
P2
P3
x1000
shRNA3
150
100
Counts
P1
P2
50
P3
250
150
200
50
100
x1000
Propidium Iodide-A
2.0
NCshRNA
shRNA4
1.5
1.0
Relative mRNA Expression
0.5
0
p21
CDK1
CDK4
CDK2
CDK6
Cyclin D1
Cyclin B1
Cyclin E
*
**
**
*
**
***
**
**
