# Supplementary material for: Heat shock protein 70-2 (HSP70-2) overexpression in breast cancer
Source: J Exp Clin Cancer Res. 2016 Sep 22;35:150. doi: 10.1186/s13046-016-0425-9 (PMC5034467; doi:10.1186/s13046-016-0425-9)
Supplement: Additional file 4: Figure S3. — HSP70-2 knockdown initiates apoptosis in breast cancer cells. (PPTX 2620 kb) [file 13046_2016_425_MOESM4_ESM.pptx]

## Slide 1
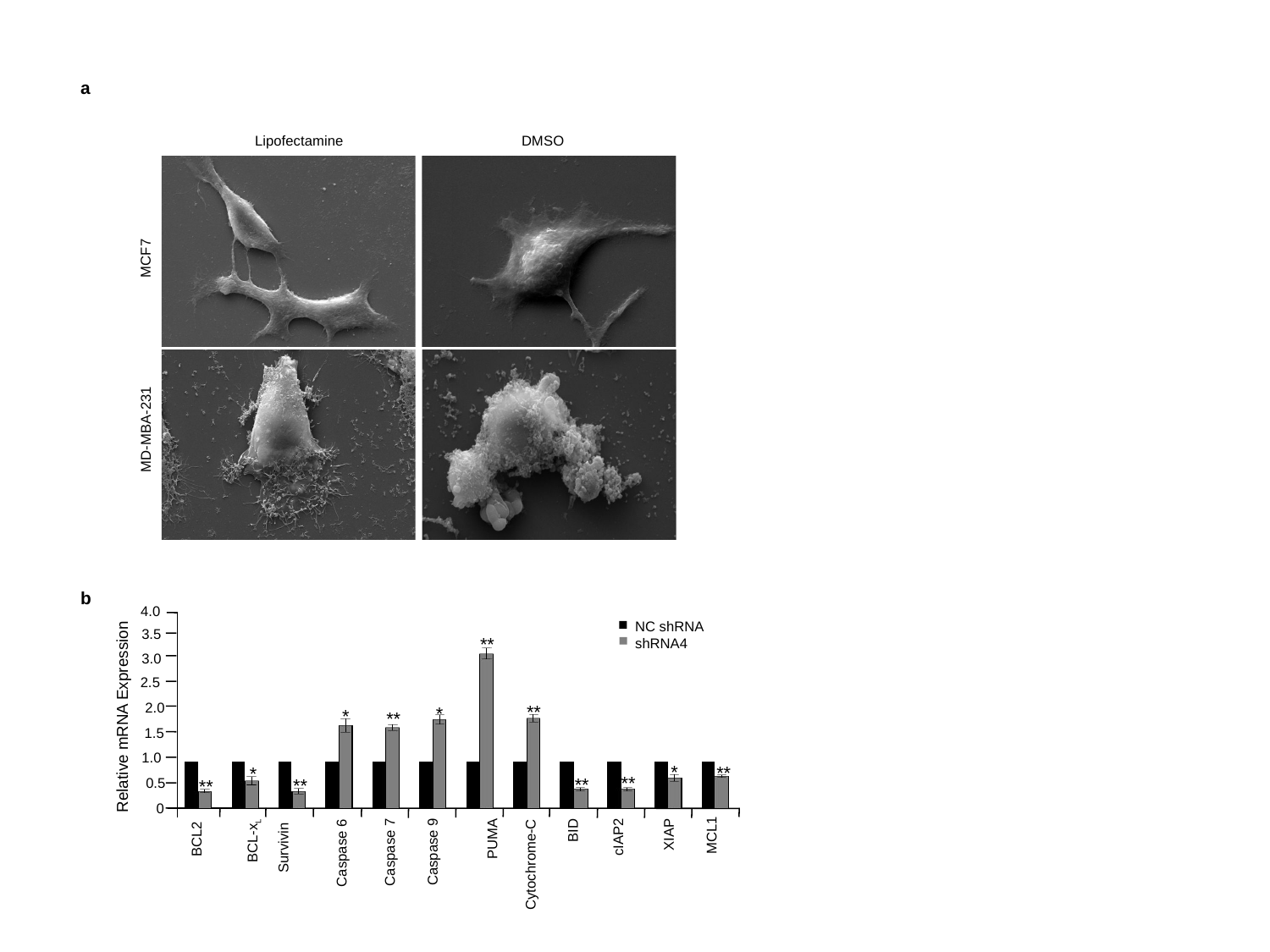

a
DMSO
Lipofectamine
MCF7
MD-MBA-231
b
4.0
NC shRNA
shRNA4
3.5
3.0
2.5
2.0
Relative mRNA Expression
1.5
1.0
0.5
0
BCL2
BID
XIAP
Caspase 6
MCL1
cIAP2
PUMA
Caspase 9
Caspase 7
BCL-xL
Survivin
Cytochrome-C
**
**
*
*
**
*
**
*
**
**
**
**
