# Supplementary material for: Heat shock protein 70-2 (HSP70-2) overexpression in breast cancer
Source: J Exp Clin Cancer Res. 2016 Sep 22;35:150. doi: 10.1186/s13046-016-0425-9 (PMC5034467; doi:10.1186/s13046-016-0425-9)
Supplement: Additional file 5: Figure S4. — Depletion of HSP70-2 in breast cancer cells inhibits cellular motility. (PPTX 274 kb) [file 13046_2016_425_MOESM5_ESM.pptx]

## Slide 1
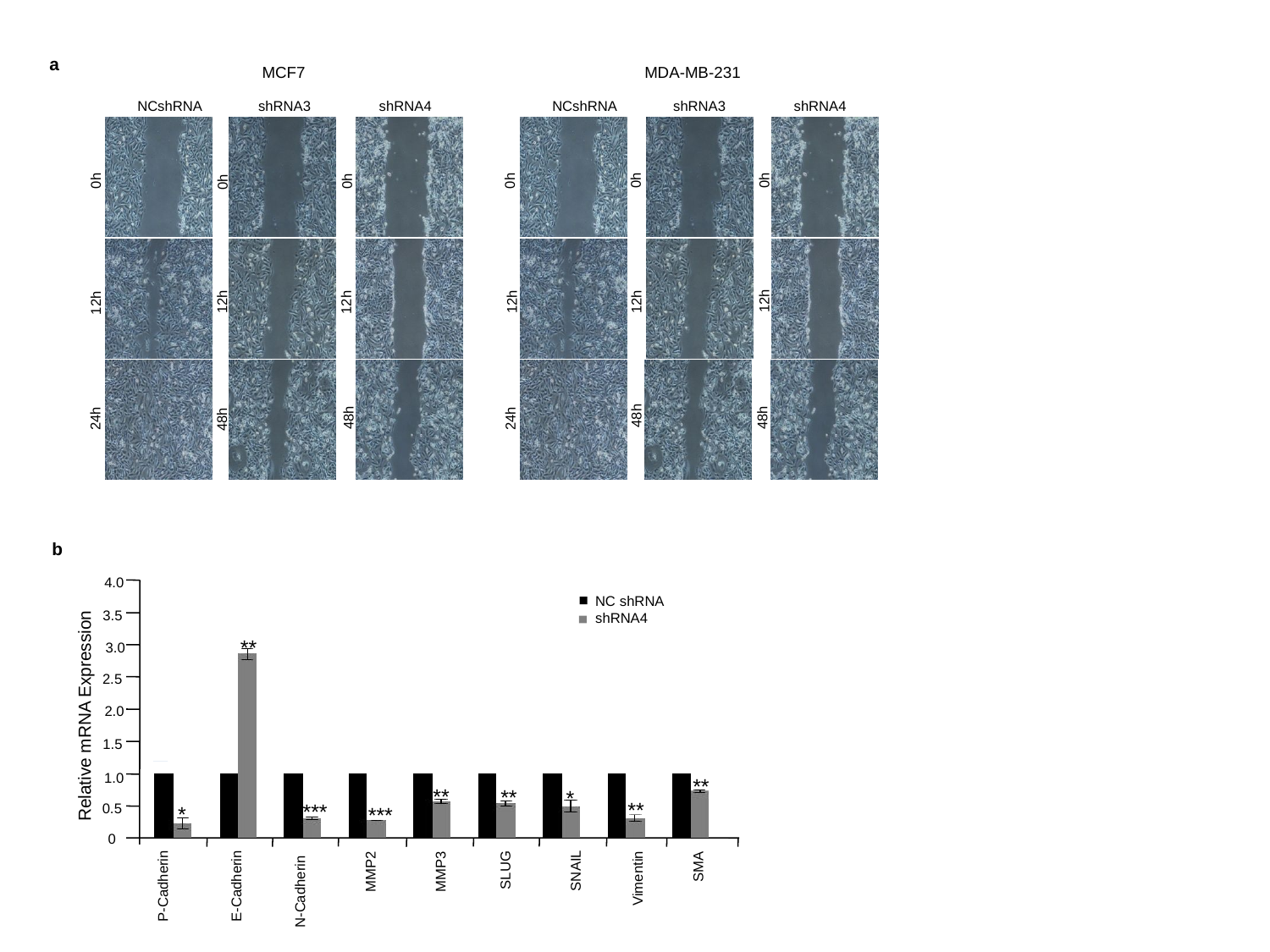

a
MCF7
MDA-MB-231
NCshRNA
shRNA3
shRNA4
0h
0h
12h
12h
12h
48h
48h
24h
0h
NCshRNA
shRNA3
shRNA4
0h
0h
0h
12h
12h
12h
48h
24h
48h
b
4.0
3.5
3.0
2.5
2.0
1.5
1.0
0.5
0
NC shRNA
shRNA4
SMA
SLUG
SNAIL
P-Cadherin
E-Cadherin
MMP3
N-Cadherin
Relative mRNA Expression
MMP2
Vimentin
**
**
**
**
*
**
***
*
***
