# Supplementary material for: Heat shock protein 70-2 (HSP70-2) overexpression in breast cancer
Source: J Exp Clin Cancer Res. 2016 Sep 22;35:150. doi: 10.1186/s13046-016-0425-9 (PMC5034467; doi:10.1186/s13046-016-0425-9)
Supplement: Additional file 6: Figure S5. — Quantitative PCR analysis of various genes involved in different signaling cascades in breast cancer tumor xenograft. (PPTX 93 kb) [file 13046_2016_425_MOESM6_ESM.pptx]

## Slide 1
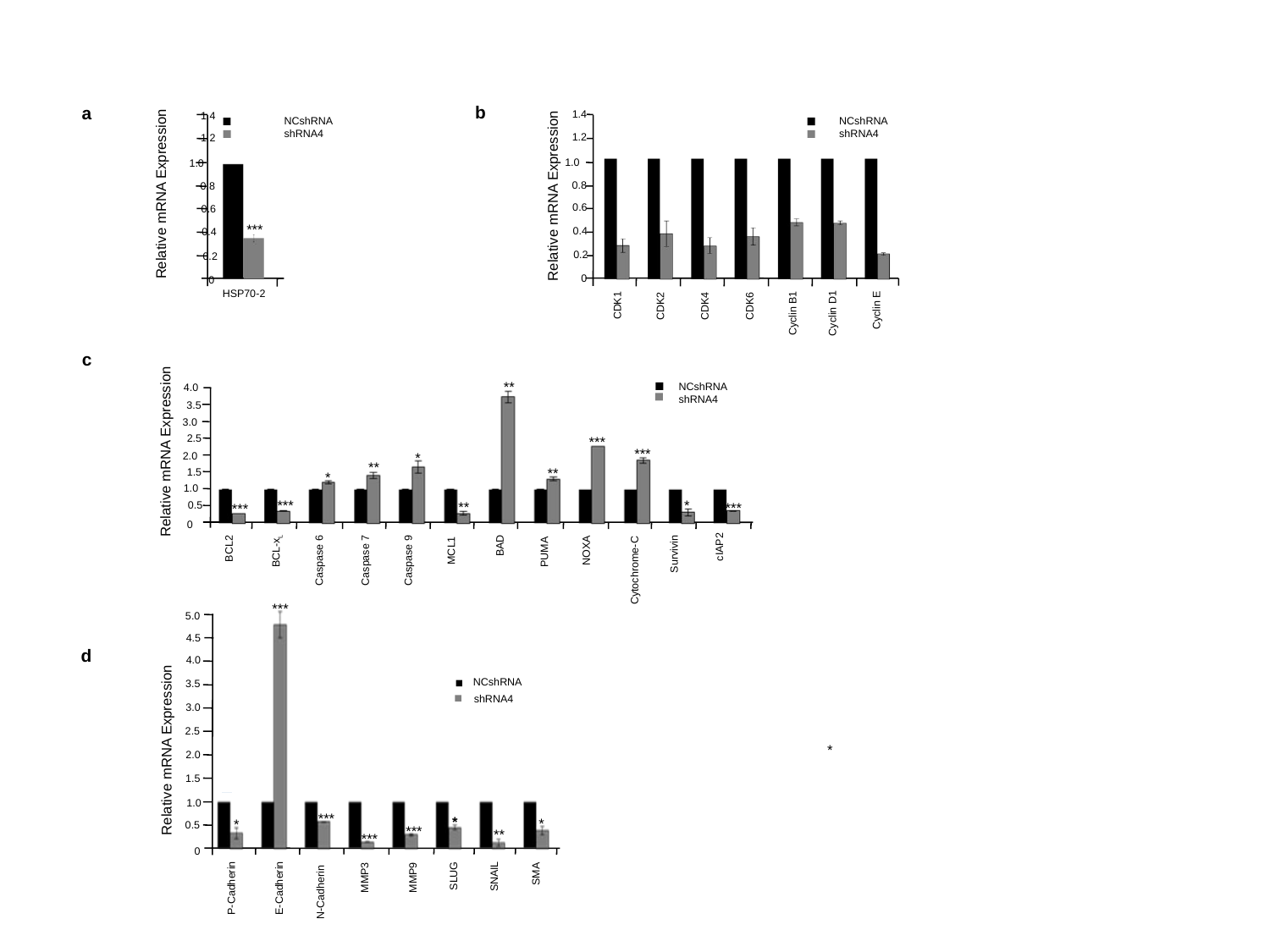

1.4
1.2
1.0
0.8
0.6
0.4
0.2
0
NCshRNA
shRNA4
Relative mRNA Expression
HSP70-2
1.4
1.2
1.0
0.8
0.6
0.4
0.2
0
NCshRNA
shRNA4
Relative mRNA Expression
CDK1
CDK2
CDK4
CDK6
Cyclin E
Cyclin D1
Cyclin B1
b
a
***
NCshRNA
shRNA4
4.0
3.5
3.0
2.5
2.0
1.5
1.0
0.5
0
Relative mRNA Expression
BAD
cIAP2
MCL1
PUMA
BCL2
Caspase 9
Caspase 7
Caspase 6
BCL-xL
Cytochrome-C
NOXA
Survivin
c
**
***
***
*
**
**
*
*
***
**
***
***
***
5.0
3.5
3.0
2.5
2.0
1.5
1.0
0.5
0
NCshRNA
MMP3
SNAIL
P-Cadherin
MMP9
SLUG
SMA
E-Cadherin
N-Cadherin
Relative mRNA Expression
shRNA4
4.5
d
4.0
*
***
*
*
*
***
**
***
